# Multifunctional AgNPs@Chitosan-g‑C_3_N_4_‑OH Nanocomposite: Efficient Catalytic Reduction of Nitro Pollutants, Antibacterial and Antioxidant Applications

**DOI:** 10.1021/acsomega.6c04143

**Published:** 2026-10-05

**Authors:** Aakhila Banu A, Abhishek Kumar, Channabasaveshwar V Yelamaggad, Bhari Mallanna Nagaraja, Alejandro Bugarin, Siddappa A. Patil

**Affiliations:** † Centre for Nano and Material Sciences, 250161 Jain (Deemed-to-be University), Jain Global Campus, Kanakapura, Ramanagaram, Bangalore 562112, India; ‡ 61921 Centre for Nano and Soft Matter Sciences, Survey No. 7, Shivanapura, Bangalore 562162, India; § Department of Chemistry & Physics, 3391 Florida Gulf Coast University, Fort Myers, Florida FL33965, United States

## Abstract

A ternary nanocomposite comprising silver nanoparticles anchored on glutaraldehyde-cross-linked chitosan supported on hydroxyl-functionalized graphitic carbon nitride (AgNPs@CS-g-C_3_N_4_-OH) was synthesized via in situ reduction of silver in a stepwise method. Structural and physicochemical characterization confirmed successful formation of the material. HR-TEM analysis showed uniformly distributed, quasi-spherical AgNPs with a lattice fringe spacing of ∼0.22 nm, corresponding to the (111) plane of crystalline silver. XPS revealed Ag(0) as the dominant species, while ICP-MS determined the silver loading to be 0.84 wt %. BET analysis indicated a mesoporous structure with surface areas of 10.75 and 7.04 m^2^ g^–1^ for CS-g-C_3_N_4_-OH and the Ag-loaded composite, respectively. The nanocomposite exhibited excellent catalytic activity in NaBH_4_-mediated reduction reactions. Conversion efficiencies of 99.79%, 97.91%, and 95.93% were achieved for 4-nitrophenol, metronidazole, and nitrofurantoin within 8, 6, and 2.5 min, respectively. The reactions followed pseudo-first-order kinetics with rate constants of 0.9871, 0.9819, and 0.9774 min^–1^, and half-lives of ∼0.70 min. Antibacterial studies showed strong activity against *Aeromonas hydrophila* and *Enterococcus faecalis*, with inhibition zones up to 16.45 ± 0.35 mm and 17.10 ± 0.70 mm at 500 μg mL^–1^. The MIC_50_ values were 28.45 and 13.40 μg mL^–1^, respectively. The material also exhibited moderate antioxidant activity with an IC_50_ of 104.6 μg mL^–1^ and 71.09% scavenging efficiency.

## Graphical abstract

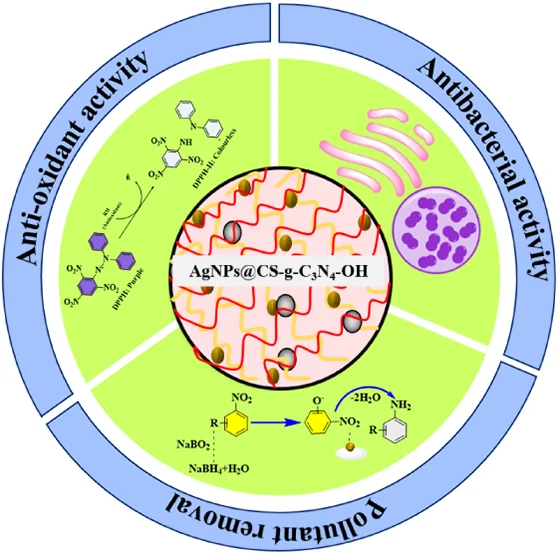

## Introduction

The rapid and continuous influx of toxic organic pollutants from pharmaceutical, agricultural, and industrial sectors into aquatic ecosystems poses a significant challenge for contemporary environmental remediation efforts. Among the wide range of hazardous organic contaminants, 4-nitrophenol (4-NP) is frequently detected in effluents from pharmaceutical and textile industries, where it is extensively used as a precursor in the manufacture of dyes, pesticides, herbicides, and paints as well as a corrosion inhibitor and pH indicator.^1^ Its persistence in both surface and groundwater systems poses serious ecological risks and threatens human health due to its toxic, mutagenic, and potentially carcinogenic nature. Consequently, the catalytic reduction of 4-NP to 4-aminophenol (4-AP), a comparatively less toxic and industrially valuable compound, has attracted considerable attention, particularly using sodium borohydride (NaBH_4_) as a reducing agent in the presence of metal catalysts.^1,2^

In addition to 4-NP, nitro-containing pharmaceutical compounds, such as metronidazole (MND) and nitrofurantoin (NFT), represent an important class of emerging micropollutants. These antibiotics are widely used and are often incompletely removed during conventional wastewater treatment processes, leading to their continuous accumulation in aquatic environments.^3^ Their persistence contributes to ecological toxicity and the proliferation of antimicrobial resistance, raising significant environmental and health concerns.^4,5^ The chemical stability and structural complexity of these nitroaromatic and nitroheterocyclic compounds make their efficient degradation particularly challenging.^5^ Traditional treatment methods, including biological processes, advanced oxidation, adsorption, and incineration, often suffer from limitations such as low efficiency, high operational cost, and the generation of secondary pollutants.^5^ Therefore, the development of efficient, stable, and recyclable catalytic systems capable of addressing a broad spectrum of nitro compounds under mild conditions is highly desirable.

Noble metal nanoparticles, especially silver nanoparticles (AgNPs), have emerged as highly effective catalysts for the reduction of nitroaromatic and nitro-antibiotic pollutants due to their high surface area and excellent electron transfer capabilities.^6^ The reduction process typically follows a Langmuir–Hinshelwood mechanism, where both the hydride donor (BH_4_^–^) and the nitro compound are adsorbed onto the catalyst surface, enabling efficient electron relay.^7^ However, unsupported AgNPs tend to aggregate and undergo Ostwald ripening, resulting in reduced catalytic activity and poor recyclability.^8^ To overcome these limitations, immobilization of the AgNPs onto a suitable support material has been widely explored to enhance stability, dispersion, and reusability.

Biopolymer-based supports, particularly chitosan, have gained considerable attention due to their biodegradability, nontoxicity, and abundance of functional groups such as amino and hydroxyl moieties, which facilitate strong coordination with metal nanoparticles.^9^ Cross-linking chitosan with glutaraldehyde further improves its mechanical strength, chemical stability, and resistance to dissolution under varying pH conditions, making it a suitable platform for heterogeneous catalysis.^10^ Such polymeric matrices not only stabilize metal nanoparticles but also enhance their accessibility and facilitate catalyst recovery.^11^

To further improve the catalytic efficiency, the incorporation of two-dimensional materials, such as graphitic carbon nitride (g-C_3_N_4_), has proven highly beneficial. g-C_3_N_4_ is a metal-free, nitrogen-rich semiconductor with a layered structure, excellent chemical stability, and favorable electronic properties.^12^ Surface functionalization, particularly hydroxyl modification (g-C_3_N_4_-OH), enhances dispersibility in aqueous media, increases the number of active sites for nanoparticle anchoring, and promotes interfacial electron transfer.^13^ The synergistic integration of AgNPs with functionalized g-C_3_N_4_ and chitosan-based matrices can significantly improve catalytic activity by increasing active site accessibility and facilitating charge transfer processes.^14^

Beyond catalytic remediation, the multifunctional properties of such nanocomposites extend into biomedical applications, particularly in the antimicrobial and antioxidant domains. Silver-based nanomaterials are well-known for their broad-spectrum antibacterial activity against both Gram-negative and Gram-positive bacteria.^15^ Waterborne pathogens such as *Aeromonas hydrophila* are responsible for severe infections in aquatic organisms and immunocompromised humans, and their increasing resistance to conventional antibiotics necessitates alternative treatment strategies.^16^ Similarly, *E. faecalis*, a Gram-positive opportunistic pathogen, is associated with persistent infections and exhibits strong resistance due to biofilm formation.^17^ AgNPs exert antibacterial effects through multiple mechanisms, including membrane disruption, intracellular penetration, interaction with biomolecules, and generation of reactive oxygen species.^18^ When combined with chitosan, which possesses inherent antimicrobial properties due to its cationic nature, a synergistic effect is achieved, enhancing the overall antibacterial efficacy.^19^

Silver nanoparticle composites based on chitosan, g-C_3_N_4_, and other carbon nitride materials have been widely investigated for catalytic and antimicrobial applications. However, comparisons of these reported systems show that the discussion of key factors such as surface area, surface leaching of the active metal nanoparticles, and activity normalized to the amount of catalyst is often limited. In our previous study, the Ag@CS-BCN nanohybrid used the mild reducing agent ascorbic acid for the reduction of silver, and the prepared nanohybrid showed rapid organic pollutant degradation and promising antibacterial and anticancer activities.^20^ However, it has a relatively low surface area of 1.7 m^2^g^–1^, and the leaching study was not reported. In the present study, the reduction occurs in situ, without the use of an additional reducing agent, and we have introduced hydroxyl groups onto the surface of g-C_3_N_4_ to provide the extra binding sites for AgNPs, improve their dispersion, and potentially reduce silver leaching. Hence, the significance of the present study lies in the improved synthesis strategy rather than claiming that the ternary nanocomposite itself is entirely novel.

In this context, the present work reports the synthesis and comprehensive characterization of AgNPs anchored on glutaraldehyde-cross-linked chitosan supported on hydroxyl-functionalized graphitic carbon nitride (AgNPs@CS-g-C_3_N_4_-OH nanocomposite). The ternary composite integrates the coordinative ability of chitosan, the structural robustness imparted by cross-linking, and the high surface area and electron-rich framework of g-C_3_N_4_-OH to produce a stable and reusable nanocatalyst. Its performance is evaluated for the catalytic reduction of representative nitro compounds, 4-NP, MNZ, and NFT, using NaBH_4_ as the reducing agent. In addition, the antibacterial activity against *Aeromonas hydrophila* (*A. hydrophila*) and *Enterococcus faecalis* (*E. faecalis*), along with antioxidant capacity assessed through the agar well diffusion method and DPPH assays, is investigated. The results demonstrate that the synergistic interaction among the composite components leads to enhanced catalytic, antibacterial, and antioxidant performance, highlighting its potential for integrated environmental and biomedical applications.

## Materials and Methods

### Materials

Melamine, chitosan (CS) powder, glacial acetic acid (AcOH), glutaraldehyde (GA), sodium hydroxide (NaOH), concentrated nitric acid (HNO_3_), concentrated sulfuric acid (H_2_SO_4_), and silver nitrate (AgNO_3_) were obtained from Sigma-Aldrich, SDFCL, and Hyma Synthesis. Nitroaromatic compounds, which include 4-nitroanisole, 4-nitrotoluene, 4-nitrobenzyl alcohol, 2-nitrobenzyl alcohol, 4-nitrobenzaldehyde, 2-nitroaniline, 4-nitroaniline, 3-nitroacetophenone, 2,4-dinitrophenol, and 2,4-dinitroaniline, were purchased from the same suppliers. The antibiotics, such as nitrofurantoin (NFT) and metronidazole (MND), were purchased from a local medical shop in Bangalore, Karnataka, India. Sodium borohydride (NaBH_4_), ethanol (99.9%), methanol, and distilled water were used without further purification unless otherwise stated. 2,2-Diphenyl-1-picrylhydrazyl (DPPH), ascorbic acid, nutrient agar, nutrient broth, and gentamicin were procured from Hi-Media Laboratories, India. Laboratory glassware and consumables, including Petri dishes, test tubes, beakers, and conical flasks, were purchased from Borosil, India. The bacterial strains *A. hydrophila* (MTCC-7966) and *E. faecalis* (MTCC-439) were obtained from the Microbial Type Culture Collection (MTCC), Chandigarh, India. Sterile 96-well plates were supplied by Tarsons Products Pvt., Ltd., India.

### Methods

#### Instrumentation

Fourier transform infrared (FT-IR) spectra were recorded using a PerkinElmer Spectrum 3. The crystalline structure and phase purity were analyzed by powder X-ray diffraction (PXRD) using a Rigaku Ultima-IV diffractometer. The surface morphology and elemental compositions were investigated using field-emission scanning electron microscopy (FE-SEM) and energy-dispersive X-ray spectroscopy (EDS) with a JEOL JSM-7100F instrument. Transmission electron microscopy (TEM) and high-resolution TEM (HR-TEM) images were obtained using a JEOL JEM-2100 microscope. To quantify the metals present on the prepared nanocomposite, Inductively Coupled Plasma Mass Spectrometry (ICP-MS) was used. The surface elemental composition and chemical states of the prepared sample were investigated using X-ray Photoelectron Spectroscopy (XPS) with a Thermo Scientific Nexsa XPS System. The instrument was operated with monochromatic Al Kα radiation (1486.6 eV) as the excitation source at a power of 72 W. All measurements were performed under ultrahigh vacuum conditions of approximately 10^–8^ mbar to ensure accurate detection of photoelectrons. Survey spectra were recorded within a binding energy range of 0–1350 eV using a pass energy of 200 eV and a dwell time of 10 ms. The system is equipped with a hemispherical electron energy analyzer, which allows precise measurement of the kinetic energy of emitted electrons. The work function of the analyzer was 4.2 eV. The surface area of the samples was determined by nitrogen adsorption–desorption measurements using the Brunauer–Emmett–Teller (BET) method on a Microtrac BELSORP MAX analyzer. Thermogravimetric analysis (TGA) was performed on a PerkinElmer STA 6000 TG/DTA Instruments under a nitrogen atmosphere in the temperature range of 50–900 °C with a heating rate of 10 °C min^–1^. The catalytic activity of the synthesized AgNPs@CS-g-C_3_N_4_-OH nanocomposite was evaluated using a UV–visible spectrophotometer (UV-1900i, Shimadzu, Japan).

#### Synthesis of g-C_3_N_4_-OH

Initially, 10 g of white melamine powder was placed in a fireclay alumina crucible and subjected to calcination at 500 °C for 4 h under a heating rate of 5 °C min^–1^. Upon completion of the calcination process, the furnace was allowed to cool naturally to ambient temperature. The resulting yellow-colored graphitic carbon nitride (g-C_3_N_4_) was obtained with a yield of 5.3891 g. The product was subsequently finely ground and preserved in an airtight container for further use.

For surface functionalization, 4 g of the synthesized g-C_3_N_4_ was dispersed in a concentrated acid mixture of HNO_3_ and H_2_SO_4_ (1:1 v/v, 40 mL) and magnetically stirred at room temperature for 2 h. The suspension was then ultrasonicated for an additional 30 min at 50 W and 40 kHz. The resulting solid was thoroughly washed with distilled water (50 mL × 2) followed by ethanol (30 mL × 2) until a neutral pH was achieved, and it was kept in a hot air oven for drying at 50 °C for 12 h. After drying, 2.1072 g of hydroxylated g-C_3_N_4_ (g-C_3_N_4_-OH) was obtained as a white powder. The synthesis procedure was adapted in accordance with previously reported literature.^21^

#### Synthesis of Silver Nanoparticles (AgNPs) Anchored on g-C_3_N_4_-OH and Cross-Linked Chitosan with Glutaraldehyde (AgNPs@CS-g-C_3_N_4_-OH Nanocomposite)

Pale-yellow chitosan powder (2 g) was initially dissolved in 2% (v/v) acetic acid (200 mL) under continuous magnetic stirring at room temperature for 30 min to obtain a homogeneous polymeric solution. Upon complete dissolution, the previously synthesized g-C_3_N_4_-OH (0.8 g) was introduced into the chitosan solution, and the suspension was further stirred for 4 h at ambient temperature to ensure uniform dispersion and effective interaction between the hydroxylated g-C_3_N_4_ and the chitosan matrix. Then, 25% glutaraldehyde (4 mL) was added as a cross-linker, and the mixture was stirred for about 2 h. Subsequently, a NaOH solution (1.5 M, 200 mL) was prepared by mixing distilled water and methanol in a 1:1 ratio. From this solution, 30 mL was taken, and AgNO_3_ (0.17 g) was dissolved in it. The resulting solution was added dropwise to the reaction mixture under constant stirring. Finally, the remaining NaOH solution (1.5 M, 170 mL) was introduced as a precipitating agent to facilitate the in situ formation of silver nanoparticles (AgNPs) within the composite matrix. The reaction was stirred for an additional 12 h at room temperature to ensure completion. The resulting product was collected by centrifugation and thoroughly washed with distilled water (40 mL × 5) from pH 14 until neutral, followed by washing with ethanol (30 mL × 2). The washed material was dried in a hot-air oven at 80 °C for 12 h. The final AgNPs@CS-g-C_3_N_4_-OH nanocomposite was obtained as a dark gray powder with a yield of 2.4532 g.

#### General Procedure for the Reduction of Nitro-Based Compounds Using AgNPs@CS-g-C_3_N_4_-OH Nanocomposite as a Nanocatalyst

The catalytic reduction of nitroarenes was carried out by stirring freshly prepared aqueous NaBH_4_ (0.4 M, 10 mL) with aqueous nitroarene (1 mM, 10 mL) under mild stirring. Then, the AgNPs@CS-g-C_3_N_4_-OH nanocomposite (14.02 mol %) was introduced to the stirring mixture. The progress of the reaction was tracked using UV–visible spectroscopy by recording the absorbance periodically. At specified time intervals, an aliquot of the reaction mixture was withdrawn, diluted with distilled water, and analyzed to determine substrate conversion. After the reaction was complete, the AgNPs@CS-g-C_3_N_4_-OH nanocomposite was isolated by centrifugation.

For the reduction of the nitro-containing antibiotics nitrofurantoin and metronidazole, the same procedure was followed at a substrate concentration of 1 mM. However, the catalyst loading was adjusted to 11.68 mol % for nitrofurantoin and 14.02 mol % for metronidazole, while the NaBH_4_ concentration was maintained at 0.4 M. All other reaction conditions were the same.

#### Procedure for the Antimicrobial and Its Minimum Inhibitory Concentration (MIC)

Antimicrobial activity was evaluated against *A. hydrophila* and *E. faecalis* using the agar well diffusion method. Nutrient agar medium was prepared by dissolving 2.8 g of commercially available nutrient agar in 100 mL of distilled water and sterilized by autoclaving at 121 °C and 15 lbs pressure for 15 min. The sterile medium was poured into Petri plates and allowed to solidify. A 24 h bacterial culture adjusted to 0.5 OD (McFarland standard) was spread on the agar plates. Wells were made in the agar, and different concentrations of the test samples (CS-g-C_3_N_4_-OH and AgNPs@CS-g-C_3_N_4_-OH nanocomposite), 50–500 μg mL^–1^, were added. The plates were incubated at 37 °C for 24 h, and antibacterial activity was determined by measuring the diameter of the inhibition zones. Gentamicin was used as a positive control, and the results were analyzed using the software GraphPad Prism 6.0.

The minimum inhibitory concentration (MIC) of the test samples was determined using the broth microdilution technique in sterile 96-well microtiter plates. The bacterial strains *A. hydrophila* and *E. faecalis* were used in the study, and nutrient broth served as the growth medium. A bacterial suspension equivalent to a 0.5 McFarland standard was prepared and diluted (1:100) in nutrient broth to obtain a final concentration of approximately 1.5 × 10^6^ CFU mL^–1^. In each well of the microtiter plates, 50 μL of the bacterial suspension was added to 50 μL of the test sample containing serially diluted concentrations (500–0.9 μg mL^–1^). Gentamicin was used as the positive control. The plates were incubated at 37 °C for 18–24 h. After incubation, bacterial growth was assessed by measuring absorbance at 600 nm with a microplate reader and by monitoring changes in turbidity. All experiments were carried out in triplicate to ensure reliable results.

#### Procedure for Antioxidant Activity

The free-radical scavenging activity of the sample was evaluated using the DPPH assay. A 0.1 mM DPPH solution was prepared in methanol, and 100 μL of this solution was mixed with 300 μL of the AgNPs@CS-g-C_3_N_4_-OH nanocomposite (labeled as AS-C) sample at various concentrations (10 to 500 μg mL^–1^). The reaction mixtures were vigorously shaken and incubated at room temperature for 30 min to allow the reaction to occur. Following incubation, the absorbance was measured at 517 nm using a UV–visible spectrophotometer, with ascorbic acid used as the reference standard. A reduction in absorbance indicates an increased free-radical scavenging ability of the sample. The percentage inhibition values and IC_50_ were subsequently determined using GraphPad Prism 6.0 software (USA). The DPPH scavenging effect was calculated using the following equation.

1 $$\%\;inhibition=\frac{\mathrm{absorbance}\;\mathrm{of}\;\mathrm{control}-\mathrm{absorbance}\;of\;\mathrm{reaction}\;\mathrm{mixture}}{absorbance\;of\;control}\times 100$$

## Results and Discussion

### Synthesis of AgNPs@CS-g-C_3_N_4_-OH

The fabrication of AgNPs@CS-g-C_3_N_4_-OH nanocomposite was done in 3 steps as displayed in Scheme 1: (i) synthesis of g-C_3_N_4_ **{1}**, (ii) surface functionalization of g-C_3_N_4_(g-C_3_N_4_-OH) **{2}**, and (iii) finally, AgNPs@CS-g-C_3_N_4_-OH nanocomposite **{3}**. First, g-C_3_N_4_ was prepared by calcining melamine in an alumina crucible at 500 °C for 4 h with a ramp rate of 5 °C min^–1^. After heating, the sample was allowed to cool naturally to room temperature. Later, it was collected and ground using a mortar and pestle. Further, the surface functionalization was carried out by treating g-C_3_N_4_ with Conc. HNO_3_ and H_2_SO_4_ in a 1:1 ratio. The acid treatment introduces a hydroxyl group (-OH) onto the surface of g-C_3_N_4_, improving its hydrophilicity^22^ and providing active sites for further interaction with polymeric matrices.^23^ For the synthesis of the final nanocomposite, initially, chitosan was dissolved in 2% acetic acid under standard laboratory conditions. Into the chitosan solution, g-C_3_N_4_-OH was added slowly and allowed to mix for another 4 h. The interaction between chitosan and g-C_3_N_4_-OH occurs mainly through hydrogen bonding and electrostatic interactions between the amino groups (−NH_2_) of chitosan and a hydroxyl group (-OH) present on the g-C_3_N_4_ surface.^24^ Then, the cross-linking agent, 25% glutaraldehyde solution, was added to the stirring mixture and allowed to stir for another 2 h. Glutaraldehyde acts as a bifunctional linker, forming Schiff base linkages (−C=N−) through a condensation reaction between the aldehyde groups (−CHO) of glutaraldehyde and the primary amine groups (−NH_2_) of chitosan, resulting in a stable cross-linked polymer network that immobilizes the g-C_3_N_4_-OH sheets within the matrix.^20,25^ Subsequently, aqueous AgNO_3_ was added dropwise to the above mixture under continuous stirring. The Ag^+^ ions interact with the functional groups present in the matrix, where electrostatic attraction and coordination occur between Ag^+^ ions and amino (−NH_2_) and hydroxyl (-OH) groups of chitosan, as well as the O/N sites of g-C_3_N_4_-OH.^26,27^ These functional groups act as binding sites as well as mild reducing sites, facilitating the *in situ* reduction of Ag^+^ to Ag^0^ and their uniform immobilization within the CS-g-C_3_N_4_-OH matrix.^26^ Lastly, the precipitating agent, aqueous NaOH, was added to the mixture, and the reaction was continued under stirring for another 12 h at room temperature. The addition of NaOH neutralizes the acetic acid and facilitates the precipitation of the cross-linked AgNPs@CS-g-C_3_N_4_-OH nanocomposite structure. The precipitate was subsequently collected in a centrifuge tube and washed with distilled water until a neutral pH was obtained, followed by washing with ethanol. Further, the sample was dried in a hot air oven, ground, and stored in an airtight container for later use.

**Scheme 1:**
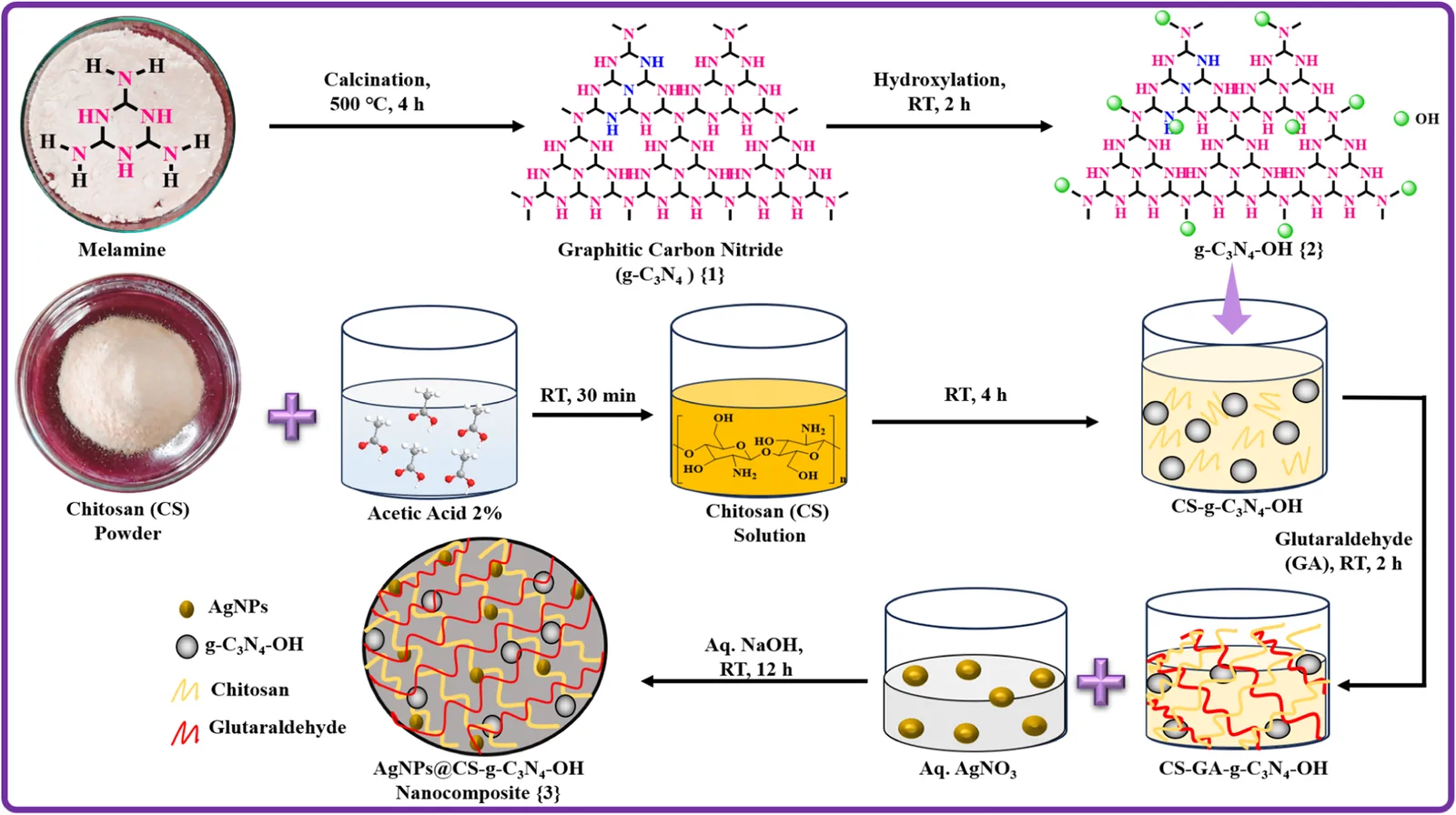
Incorporation of AgNPs into Chitosan Crosslinked with Glutaraldehyde and g-C_3_N_4_-OH Matrix to Synthesize AgNPs@CS-g-C_3_N_4_-OH Nanocomposite

### FT-IR and PXRD Analyses

The FTIR spectra of a support material (CS-g-C_3_N_4_-OH) and a final AgNPs@CS-g-C_3_N_4_-OH nanocomposite are shown in Figure 1a. In the FTIR spectrum of the CS-g-C_3_N_4_-OH composite shown in Figure 1ai, a broad absorption band observed between approximately 2982 and 3724 cm^–1^, centered at 3435 cm^–1^, is attributed to overlapping stretching vibrations of -OH and -NH groups present in chitosan and hydroxylated g-C_3_N_4_. The extension of this band toward higher wavenumbers (around 3500 cm^–1^) indicates the presence of -OH groups, while the lower region corresponds to hydrogen-bonded -OH/-NH stretching vibrations, confirming strong intermolecular hydrogen bonding within the composite matrix.^28,29^ The absorption band in the region of ∼2924 cm^–1^ corresponds to the aliphatic C–H (−CH_2_/–CH_3_) stretching vibrations of the chitosan backbone. These peaks result from the symmetric and asymmetric stretching modes of methylene groups present in the polysaccharide structure.^28^ The characteristic band at 1638 cm^–1^ corresponds to the stretching vibrations of C=N (Schiff-base linkage) formed by the reaction between the amino groups of chitosan and glutaraldehyde, which overlaps with the amide I (C=O) vibration and skeletal stretching of the heptazine (tri-s-triazine) units of g-C_3_N_4_. The appearance of this band confirms the successful cross-linking of chitosan with glutaraldehyde. In the case of the AgNPs@CS-g-C_3_N_4_-OH nanocomposite shown in Figure 1aii, a slight shift in the intensity and position of this band is observed, which results from the interaction of chitosan chains with AgNPs and g-C_3_N_4_-OH sheets, causing minor structural rearrangements in the polymer matrix. The C=N (Schiff-base)/amide I band shifts slightly from 1638 to 1634 cm^–1^, while the amide II band appears at approximately 1546 cm^–1^, corresponding to N–H bending coupled with C–N stretching vibrations.^30^ The stretching vibrational bands observed between ∼1537–1194 cm^–1^ is assigned to the C–N and C=N of heterocyclic g-C_3_N_4_.^29^ The extended region of approximately 1194–915 cm^–1^, attributed to C–N stretching vibrations, C–O–C glycosidic stretching, and N–H bending modes, mainly originates from the g-C_3_N_4_ heterocyclic units and chitosan backbone, indicating strong interfacial interaction between the two components.^28,29^ The band observed around 1410–1243 cm^–1^ is characteristic of aromatic C–N stretching vibrations of the heptazine units of g-C_3_N_4_. The peaks observed within the range of ∼836–643 cm^–1^ correspond to the breathing mode of the tri-s-triazine ring (∼800 cm^–1^),^31^ confirming the structural integrity of g-C_3_N_4_. In the AgNPs@CS-g-C_3_N_4_-OH nanocomposite Figure 1aii, slight shifts and intensity changes are observed in the -OH/–NH, C=N (Schiff-base)/amide I, amide II, and C–N regions, indicating the involvement of these functional groups in the coordination and stabilization of AgNPs within the composite matrix.^32^

**Figure 1:**
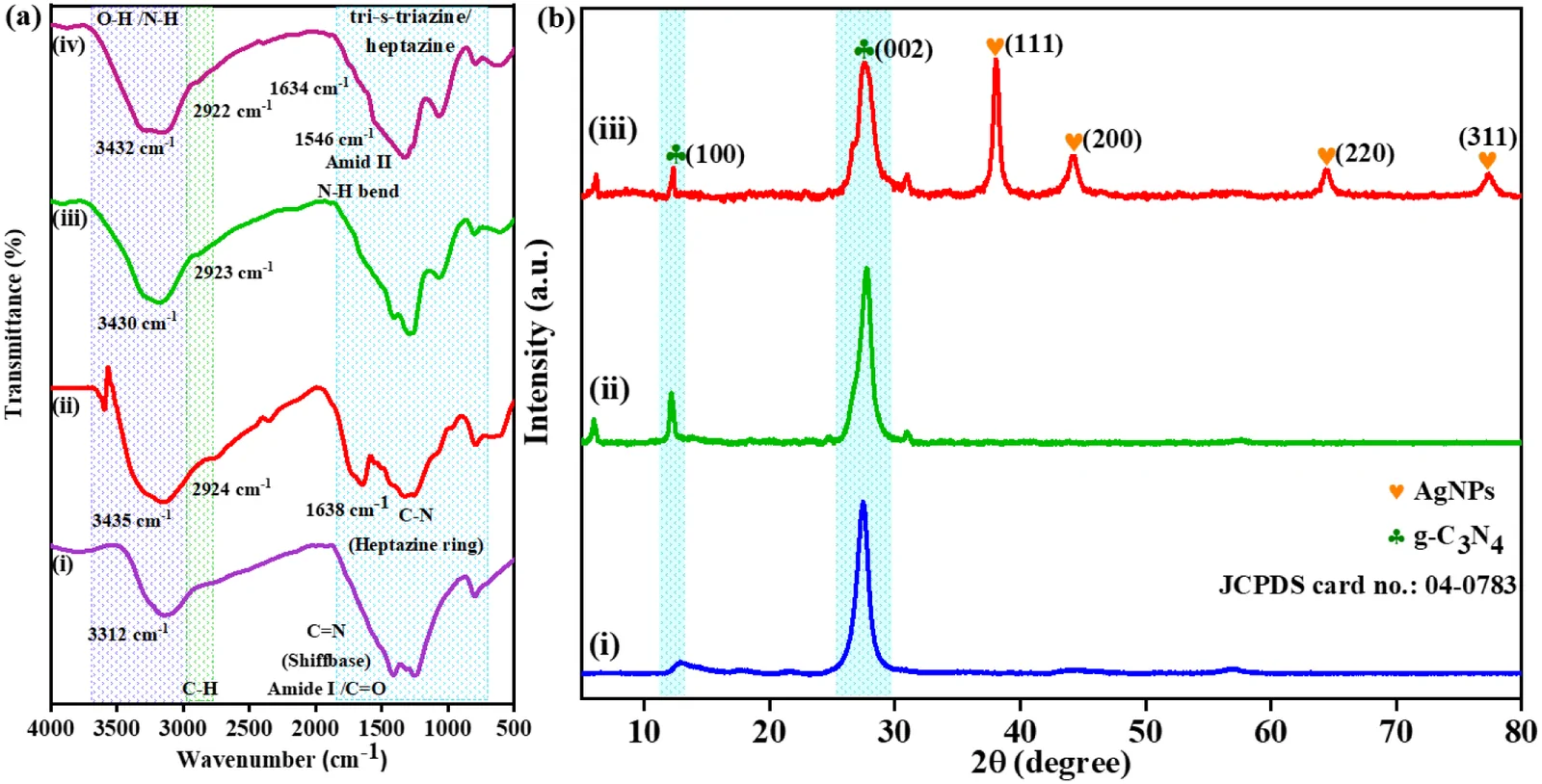
(a) FT-IR spectra of (i) g-C_3_N_4_, (ii) g-C_3_N_4_-OH, (iii) CS-g-C_3_N_4_-OH and (iv) AgNPs@CS-g-C_3_N_4_-OH nanocomposite; (b) diffraction patterns of (i) g-C_3_N_4_, (ii) CS-g-C_3_N_4_-OH, and (iii) AgNPs@CS-g-C_3_N_4_-OH nanocomposite.

Figure 1bi-iii shows the PXRD profiles obtained for g-C_3_N_4_, CS-g-C_3_N_4_-OH and AgNPs@CS-g-C_3_N_4_-OH nanocomposite. The diffraction peaks at 2θ ≈ 12.9° and 27.4° correspond to the (100) and (002) planes of g-C_3_N_4_, which represent the in-plane structural packing and interlayer stacking of the graphitic structure.^29^ In CS-g-C_3_N_4_-OH, the main peak at ∼27.4° is retained, confirming that the structure of g-C_3_N_4_ remains intact after functionalization. However, the peak becomes broader and slightly reduced in intensity, indicating the incorporation of amorphous chitosan and interaction between chitosan and g-C_3_N_4_-OH.^28^ No distinct peaks corresponding to pure chitosan are clearly observed due to its low crystallinity and overlap with g-C_3_N_4_ peaks. For the AgNPs@CS-g-C_3_N_4_-OH nanocomposite, additional peaks appear at ∼38.0°,44.3°, 64.5°, and 77.3°, which are indexed to the (111), (200), (220), and (311) planes of face-centered cubic (FCC) silver nanoparticles, confirming the successful formation of crystalline AgNPs (JCPDS No. 04–0783). A slight shift in the g-C_3_N_4_ peak suggests the interaction of AgNPs with the support material. This confirms the successful formation of the AgNPs@CS-g-C_3_N_4_-OH nanocomposite.

### FE-SEM and EDS Analyses

FE-SEM images of CS-g-C_3_N_4_-OH and AgNPs@CS-g-C_3_N_4_-OH nanocomposites are shown in Figure S1a-b. In Figure S1a, the g-C_3_N_4_-OH composite exhibits an irregular, layered morphology with some agglomeration and rough surfaces, indicating a strong interaction between the polymer-chitosan and the functionalized g-C_3_N_4_.^28,29^ After the incorporation of AgNPs, the morphology of AgNPs@CS-g-C_3_N_4_-OH, as revealed in Figure S1b shows a more uniform and compact structure with small particles on the surface, which are attributed to AgNPs. These NPs appear well-distributed, with no major aggregation, suggesting good dispersion and stabilization within the matrix. The EDS spectrum in Figure S1c of the AgNPs@CS-g-C_3_N_4_-OH nanocomposite confirms the presence of C, N, O, and Ag, indicating successful incorporation of AgNPs into the matrix. Figure S1d-h displays the elemental mapping of AgNPs@CS-g-C_3_N_4_-OH nanocomposite, showing that all the elements are uniformly distributed and that AgNPs are effectively immobilized and stabilized within the composite structure.

### HR-TEM Analyses

The morphological and structural features of the prepared CS-g-C_3_N_4_-OH and AgNPs@CS-g-C_3_N_4_-OH nanocomposite were investigated using HR-TEM and related techniques, which are shown in Figure 2a–e. Figure 2a shows the TEM image of the CS-g-C_3_N_4_-OH matrix, which exhibits a sheet-like and layered morphology with relatively smooth surfaces, characteristic of exfoliated graphitic carbon nitride integrated within the chitosan matrix. The absence of a distinct particulate contrast indicates that no metallic nanoparticles are present at this stage. In contrast, Figure 2b displays the TEM image of the AgNPs@CS-g-C_3_N_4_-OH nanocomposite. Well-dispersed dark, more or less spherical spots corresponding to AgNPs are clearly observed on the CS-g-C_3_N_4_-OH matrix support. The nanoparticles are uniformly anchored onto the CS-g-C_3_N_4_-OH matrix without significant aggregation, indicating effective stabilization by the functional groups of chitosan and the g-C_3_N_4_-OH framework. The inset further confirms the nanoscale distribution of AgNPs across the support. The HR-TEM image in Figure 2c reveals clear lattice fringes with an interplanar spacing of ∼0.22 nm, which can be indexed to the (111) crystallographic plane of face-centered cubic (fcc) silver.^33^ This confirms the crystalline nature of the deposited AgNPs. Figure 2d presents the selected area electron diffraction (SAED) pattern of the nanocomposite, showing a series of concentric diffraction rings indexed to the (111), (200), (220), and (311) planes of fcc silver (for the raw image, see Figure S2). Figure 2f shows the particle size distribution of the fresh AgNPs@CS-g-C_3_N_4_-OH nanocomposite. Particle sizes were determined by measuring 50 AgNPs, and the silver nanoparticles exhibited an average diameter of 12.82 ± 6.33 nm (mean ± SD, *N* = 50). The presence of distinct rings rather than spots indicates the polycrystalline nature of the AgNPs. The HAADF-STEM image in Figure 2e further supports the successful incorporation of AgNPs, where brighter contrast corresponds to heavier Ag atoms. The corresponding elemental mapping images demonstrate the uniform distribution of C, N, O, and Ag throughout the nanocomposite. Carbon and nitrogen signals confirm the presence of the CS-g-C_3_N_4_-OH matrix, while oxygen arises from hydroxyl functionalities. The homogeneous dispersion of Ag signals verifies the successful loading and stabilization of AgNPs on the support material.

**Figure 2:**
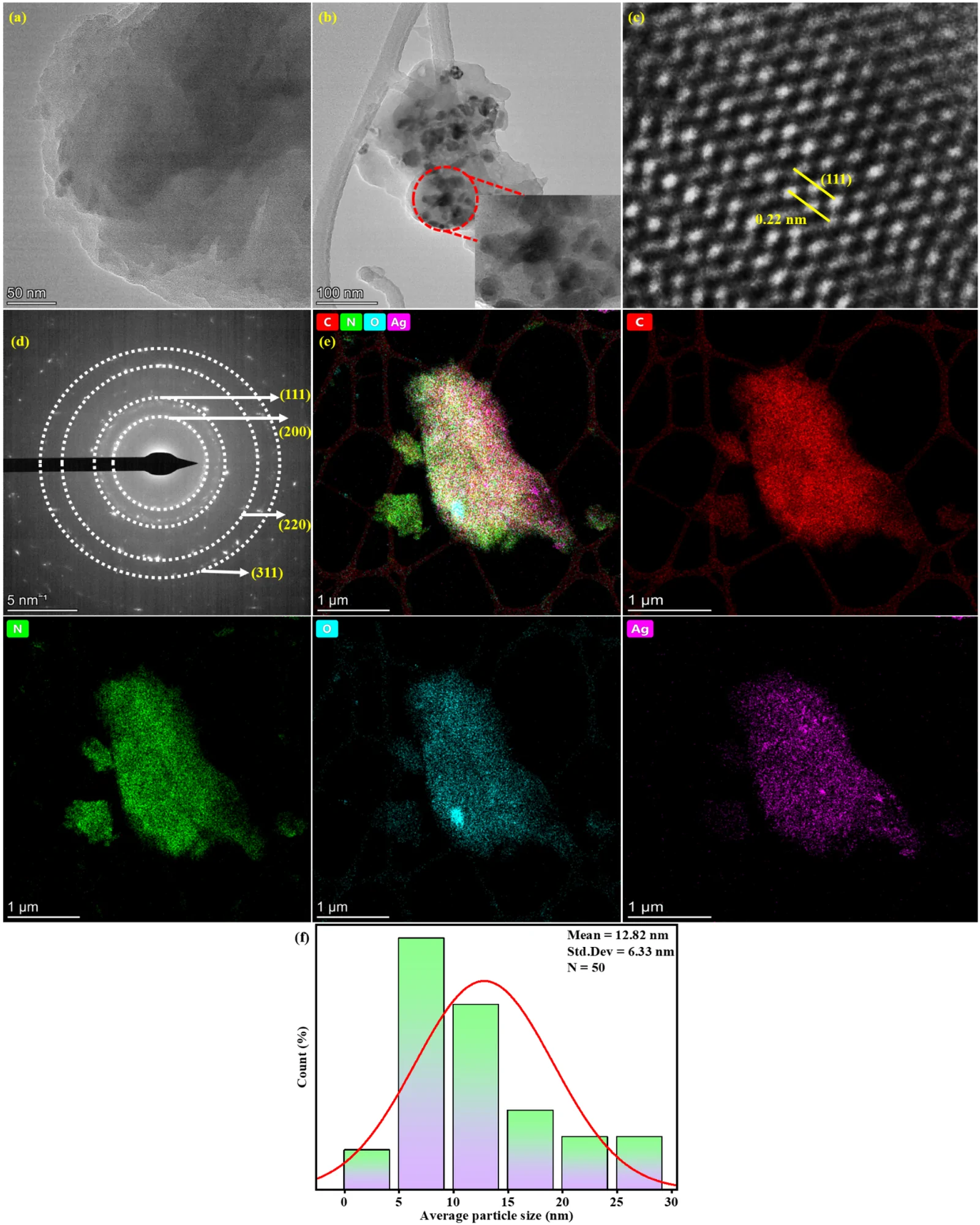
HR-TEM image of (a) CS-g-C_3_N_4_-OH, (b) AgNPs@CS-g-C_3_N_4_-OH nanocomposite, and (c) lattice fringes with *d*-spacing of AgNPs@CS-g-C_3_N_4_-OH nanocomposite; (d) SAED pattern of AgNPs@CS-g-C_3_N_4_-OH nanocomposite; (e) HAADF-STEM image of AgNPs@CS-g-C_3_N_4_-OH nanocomposite; and (f) average particle size distribution histograms of AgNPs@CS-g-C_3_N_4_-OH nanocomposite.

### XPS Analysis

The elemental composition and surface chemical states of the synthesized AgNPs@CS-g-C_3_N_4_-OH nanocomposite were analyzed by X-ray photoelectron spectroscopy (XPS), as shown in Figure 3a–e. Figure 3a shows the survey spectrum for C *1s*, Ag *3d*, N *1s*, and O *1s*, confirming the successful anchoring of metallic Ag onto the CS-g-C_3_N_4_-OH support material. Figure 3b, the O *1s* spectrum revealed multiple peaks found at ∼531.22 eV, ∼532.10 eV, and ∼533.04 eV, which indicate the presence of carbonyl oxygen (C=O), hydroxyl and ether groups (C–OH/C–O–C), and surface hydroxyl groups associated with oxygenated functionalities within the polymer matrix, chitosan backbone, and absorbed water molecules. Notably, the absence of a characteristic lattice oxygen peak corresponding to Ag_2_O suggests that Ag is not present in the form of bulk silver oxide, confirming metallic silver’s predominance within the AgNPs@CS-g-C_3_N_4_-OH nanocomposite.^34^ The N *1s* spectrum in Figure 3c displays peaks at ∼399.31 eV, 400.79, and 402.13 eV, which are attributed to sp^2^-hybridized nitrogen (C=N–C) in the triazine/heptazine units of g-C_3_N_4_, tertiary nitrogen (N-(C)_3_), or bridging nitrogen atoms, and protonated (−NH_3_^+^) or amino nitrogen species from chitosan.^35^ These results confirm the presence of nitrogen-rich functional groups and their role in stabilizing AgNPs through electron donation and coordination interactions, thereby facilitating strong metal–support interaction. The Ag spectrum exhibits two spin–orbit doublets at ∼368.2 eV (Ag 3d_5/2_) and ∼374.6 eV (Ag 3d_3/2_), confirming the presence of metallic Ag. A slight shift or additional feature around ∼367.9 eV and ∼373.9 eV suggests the presence of partially oxidized silver (Ag^+^), indicating minor surface oxidation or interaction with functional groups and coordination between Ag and the support CS-g-C_3_N_4_-OH matrix,^36,37^ and it is demonstrated in Figure 3d. In Figure 3e, the C *1s* spectrum confirms the presence of various carbon environments arising from both g-C_3_N_4_ and chitosan. The peaks detected at ∼284.5, ∼285.6, and ∼288.3 eV represent the C–C/C=C (graphitic carbon), C–N/C–O from chitosan and linked frameworks, and N–C=N/C=N of g-C_3_N_4_. These features confirm the successful integration of carbon nitride into the chitosan matrix.^38^

**Figure 3:**
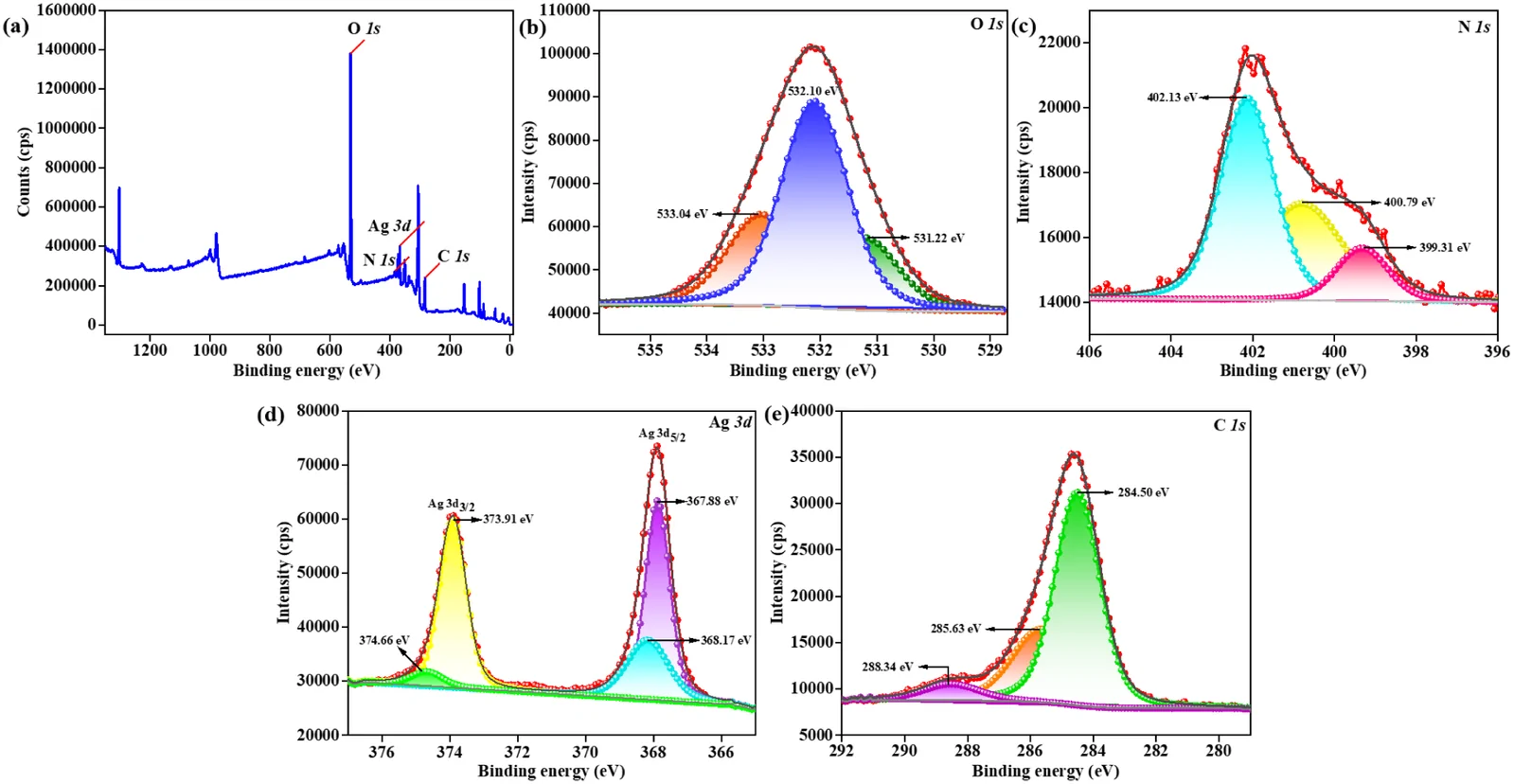
XPS spectra of (a) survey spectrum, (b) O 1s, (c) N 1s, (d) Ag 3d, and (e) C 1s.

### ICP-MS Analysis

Inductively coupled plasma-mass spectrometry (ICP-MS) was employed to quantify the Ag content in the AgNPs@CS-g-C_3_N_4_-OH nanocomposite, revealing a metal loading of 0.84 wt %.

### Thermogravimetric (TG) Analysis

The TGA profile of the AgNPs@CS-g-C_3_N_4_-OH nanocomposite is shown in Figure S3 and exhibits a stepwise weight-loss pattern, confirming its nanocomposite structure. The initial weight loss below ∼120 °C is due to the removal of physically absorbed moisture. A gradual decrease between ∼150 and 300 °C is attributed to the breakdown of weakly bound functional groups and the initial degradation of chitosan. A significant weight loss in the range of ∼300–500 °C is linked to the decomposition of the chitosan backbone, along with partial disruption of the g-C_3_N_4_ network. Beyond ∼500 °C, the curve shows a slower weight reduction, indicating the decomposition of more stable graphitic carbon nitride structures.^20,36,39^ The residual weight at elevated temperatures is due to thermally stable AgNPs, indicating their effective incorporation within the composite. Overall, the enhanced thermal stability arises from the strong synergistic interactions among chitosan, g-C_3_N_4_-OH, and AgNPs.

### BET Surface Area Analysis

The surface area and pore structure of the CS-g-C_3_N_4_-OH and AgNPs@CS-g-C_3_N_4_-OH nanocomposites were scrutinized using N_2_ adsorption–desorption measurements at a degassing temperature of 120 °C for 6 h under vacuum to remove physically adsorbed moisture and volatile species. As shown in Figure 4a and c, both materials exhibit Type IV isotherm characteristics according to the IUPAC classification, confirming the presence of mesoporous structures. A distinct hysteresis loop is observed in the intermediate-to-high relative pressure (*P*/*P*_0_) region, which can be attributed to capillary condensation within the pores. Closer inspection suggests that the hysteresis loop resembles an H3-type profile, typically associated with slit-shaped pores formed by layered or aggregated structures. This observation is consistent with the sheet-like morphology of the CS-g-C_3_N_4_-OH matrix, as evidenced from HR-TEM analysis. The BET surface area of CS-g-C_3_N_4_-OH was determined to be 10.75 m^2^ g^–1^, with a total pore volume of 0.0086 cm^3^ g^–1^ and an average pore diameter of 3.19 nm. The corresponding BJH pore size distribution in Figure 4b shows a relatively narrow distribution within the mesoporous range, indicating a fairly uniform pore structure. After the incorporation of AgNPs, as demonstrated in Figure 4d, the BET surface area changes to 7.04 m^2^ g^–1^, accompanied by a pore volume of 0.0069 cm^3^ g^–1^ and an average pore diameter of 3.93 nm. The retention of the isotherm profile suggests that the mesoporous framework remains intact after AgNP loading. However, the decrease in pore volume and pore diameter, along with the broader pore size distribution, indicates the partial occupation of pore channels and increased structural heterogeneity due to the deposition of AgNPs. The slight decrease in surface area may be attributed to the incorporation of AgNPs onto the support chitosan-g-C_3_N_4_-OH layers, and it can occupy the available surface and pore volume, thereby limiting the surface area of the supporting CS-g-C_3_N_4_-OH matrix. Overall, most of the catalytic performance of the AgNPs@CS-g-C_3_N_4_-OH nanocomposite arises from the synergistic interaction between AgNPs, CS, and g-C_3_N_4_-OH, rather than from a high-surface-area porous architecture.

**Figure 4:**
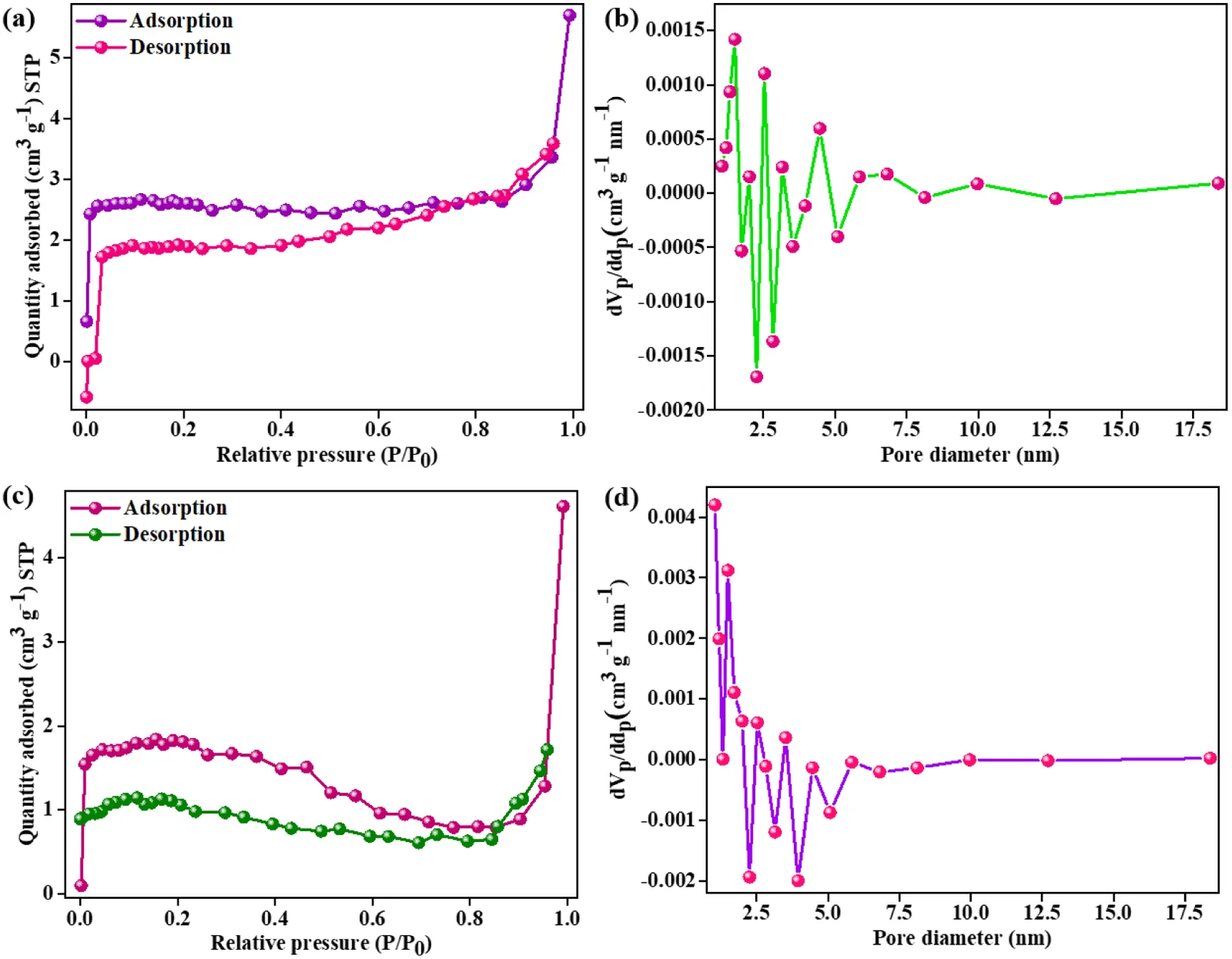
(a) N_2_ adsorption–desorption isotherm and (b) BJH pore size distribution of CS-g-C_3_N_4_-OH; (c) N_2_ adsorption–desorption isotherm and (d) BJH pore size distribution of AgNPs@CS-g-C_3_N_4_-OH nanocomposite.

### Catalytic Investigation of AgNPs@CS-g-C_3_N_4_-OH Nanocomposite as a Nanocatalyst in the Reduction of Nitroaromatics to Amines

The catalytic efficiency of the synthesized AgNPs@CS-g-C_3_N_4_-OH nanocomposite was evaluated for the reduction of nitroaromatic compounds to their corresponding amines using NaBH_4_ as a reducing agent. As displayed in Scheme 2a–c, the proposed catalytic pathway describes the transformation of nitroaromatic substrates such as 4-nitrophenol (Scheme 2a), metronidazole (Scheme 2b), and nitrofurantoin (Scheme 2c) to their corresponding aromatic amines in the presence of the prepared AgNPs@CS-g-C_3_N_4_-OH nanocomposite and aqueous NaBH_4_ under ambient reaction conditions.

**Scheme 2:**
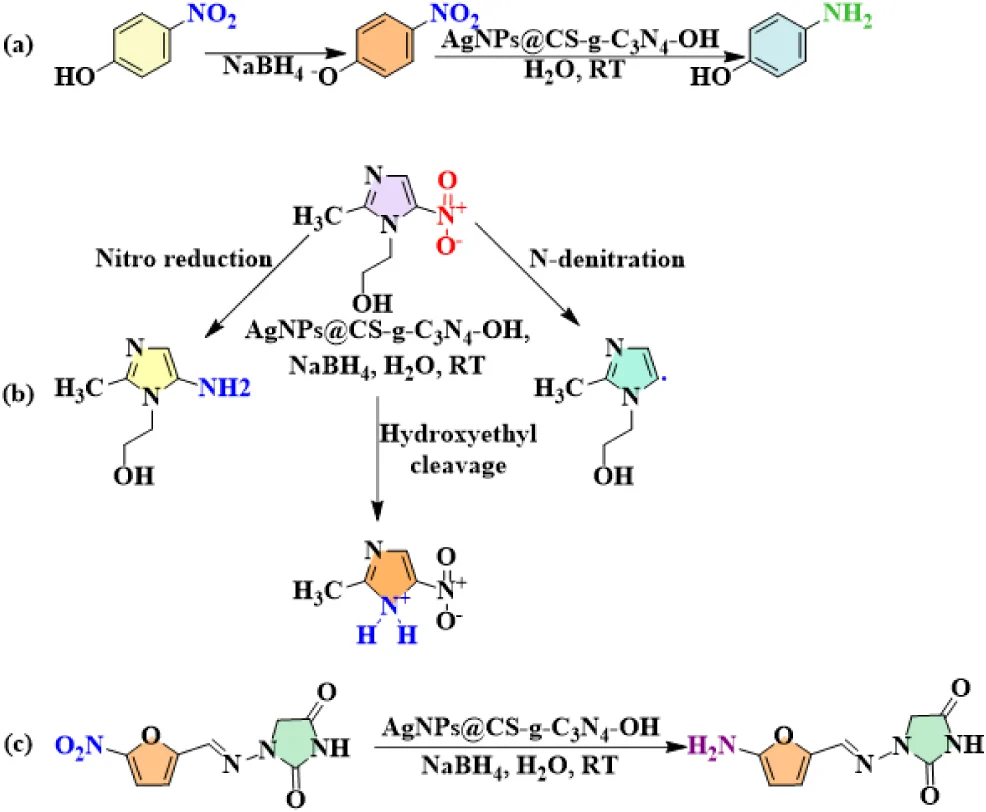
Reaction Pathway for Catalytic Reduction of Nitroaromatic Compounds over AgNPs@CS-g-C_3_N_4_-OH Nanocomposite: (a) Reduction of 4-NP to 4-AP, (b) Reduction/Degradation of Metronidazole, and (c) Reduction of Nitrofurantoin

### Kinetic Studies: Catalytic Reduction of 4-Nitrophenol and Nitro-Based Antibiotics (Nitrofurantoin and Metronidazole)

The catalytic performance of AgNPs@CS-g-C_3_N_4_-OH nanocomposite was evaluated through the reduction of nitroaromatic compounds in the presence of NaBH_4_, as illustrated in Figure 5 as a model reduction reaction. Time-dependent UV–visible absorption spectra (Figure 5a–c) show a progressive decrease in the characteristic absorption peaks as the reaction time increases, confirming the progression of the reduction process. The maximum absorption wavelengths (λ_max_) for 4-NP, MND, and NFT were observed at 400, 318, and 387 nm, respectively. The complete disappearance of these peaks indicates efficient catalytic conversion. The *C_t_*/*C*_0_ versus time (*t*) plots (Figure 5d–f) show a steady decline in concentration, further supporting the advancement of the reduction reaction. The reduction efficiencies were determined for all compounds, with 4-NP (14.02 mol %, 0.4 M), MND (14.02 mol %, 0.4 M), and NFT (11.68 mol %, 0.4 M), exhibiting conversion efficiencies of approximately 99.79%, 97.91%, and 95.93% within 8, 6, and 2.5 min, respectively. The ln­(*C*_0_/*C_t_*) versus time plots (Figure 5g–i) display excellent linearity, indicating that the reduction follows pseudo-first-order kinetics due to the excess of NaBH_4_. The kinetic data were fitted using the following equation.

2 $$\ln\left(C_{0}/C_{t}\right)=kt$$

**Figure 5:**
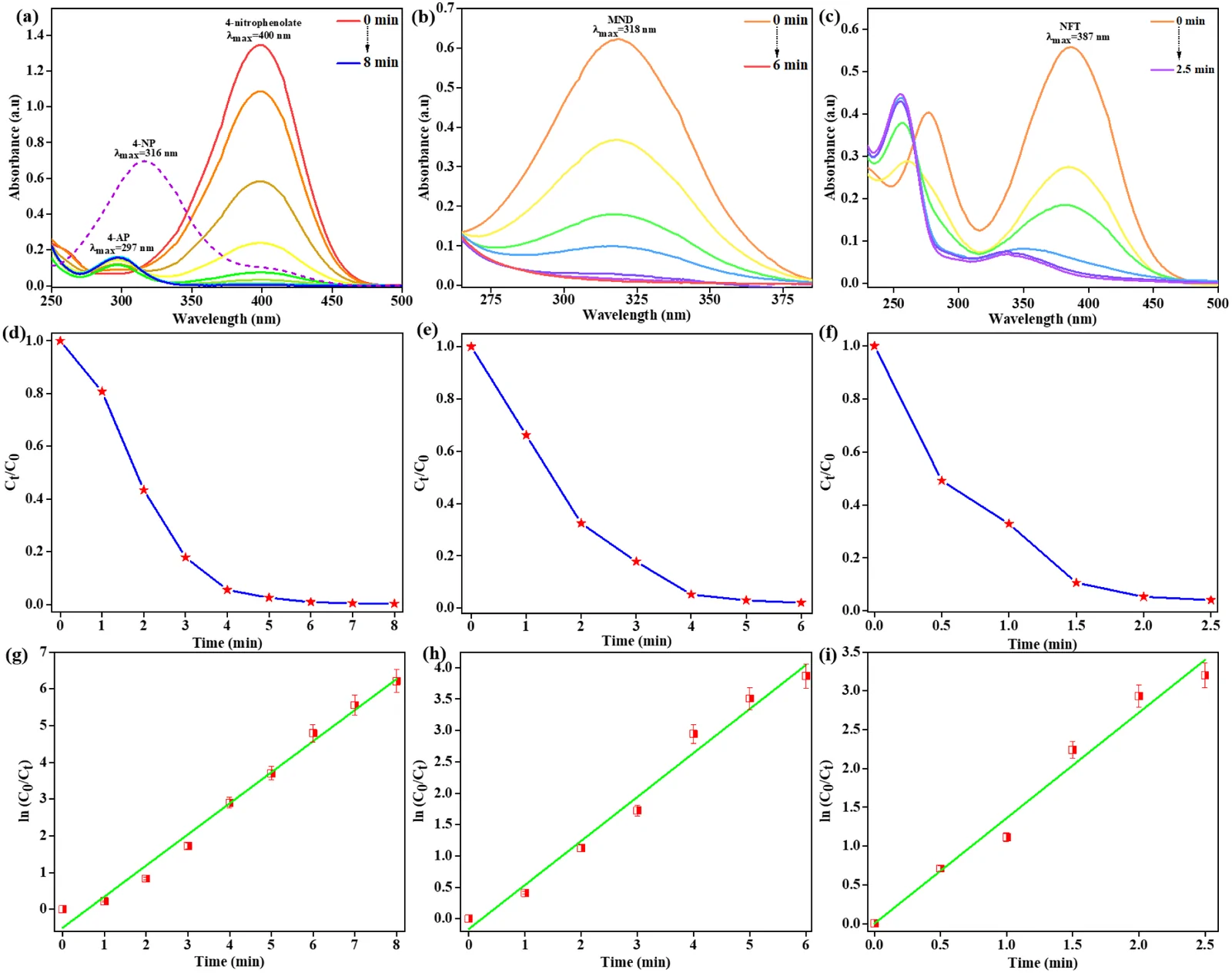
Time-dependent UV–visible absorption spectra for the catalytic reduction of (a) 4-NP, (b) MND, and (c) NFT using AgNPs@CS-g-C_3_N_4_-OH nanocomposite. (d–f) Plots of normalized concentration (*C_t_*/*C*_0_) as a function of reaction time. (g–i) Linear pseudo-first-order kinetics plots of ln­(*C*_0_/*C_t_*) versus time to determine the apparent rate constant for (d, g) 4-NP, (e, h) MND, and (f, i) NFT.

Here, *C*_0_ is the initial concentration, *C_t_* is the concentration at time *t*, and *k* is the apparent rate constant. The high correlation coefficient (*R* ^2^) values confirm the reliability of the linear fitting for all three systems. The correlation coefficients (*R* ^2^) confirm that the linear fitting is strong in all three systems. The rate constants are obtained from the slopes of the linear plots, and their values are 0.9871, 0.9819, and 0.9774 min^–1^ for 4-NP, MND, and NFT, respectively. A higher slope indicates a faster reaction rate. Accordingly, 4-NP exhibits the fastest reduction kinetics, followed by MND, while NFT shows comparatively slower kinetics. The half-life (*t*_1/2_) values were also determined. The half-life (*t*_1/2_) values were calculated using first-order kinetics, and the results show an inverse relationship between half-life and the rate constant. The calculated half-life values are 0.7020, 0.7058, and 0.7090 min for 4-NP, MND, and NFT, respectively. This trend is consistent with the observed decrease in absorption intensity in the UV–visible spectra. Control experiments were performed to verify the catalytic role of the AgNPs@CS-g-C_3_N_4_-OH nanocomposite. The UV–visible spectra were initially recorded in the absence of AgNPs@CS-g-C_3_N_4_-OH and NaBH_4_. No significant reduction was observed in the absence of NaBH_4_, and 97.03% conversion efficiency in 60 min was observed without the catalyst, confirming that both are essential for the faster reaction. Further, the control experiment using the CS-g-C_3_N_4_-OH support (without AgNPs), substrate alone, g-C_3_N_4_-OH, chitosan/glutaraldehyde matrix, and bare AgNPs for 4-NP was carried out under identical reaction conditions to confirm that the high activity arises from the synergistic effect of the material. The support CS-g-C_3_N_4_-OH did not show any visible catalytic activity toward the reduction of 4-NP, indicating that the AgNPs are the primary active catalytic sites. All experimental parameters, including catalyst loading, NaBH_4_ concentration, and corresponding conversion efficiencies, are summarized in Table 1. Overall, Figure 5 confirms that the AgNPs@CS-g-C_3_N_4_-OH nanocomposite acts as an efficient nanocatalyst.

**Table 1:** Reaction Parameters for the NaBH_4_-mediated Reduction of Nitroaromatics Were Systematically Optimized in the Presence of the AgNPs@CS-g-C_3_N_4_-OH Nanocomposite^a^

SI. No.	Substrate	NaBH_4_ (M)	AgNPs@CS-g-C_3_N_4_-OH (mol % of Ag)	Time (min)	Conversion efficiency (%)
1	4-NP (1 mM)	-	14.02	60^b^	-
2		0.4	-	60	97.03
3		0.3	7.79	12	99.55
4		0.4	7.79	13	99.50
5		0.5	7.79	12	99.70
6		0.4	9.34	12	99.18
7		0.4	11.68	12	99.63
8		0.4	14.02	8	99.79
9		0.4	15 mg^c^	15^b^	-
10		-	-	60^b^	-
11		0.4	18 mg^d^	8	47.83
12		0.4	18 mg^e^	8	54.52
13		0.4	0.16 mg^f^	8	44.28
14	4-NP (1.5 mM)	0.4	14.02	10	99.67
15	4-NP (0.5 mM)	0.4	14.02	7	98.89
16	MND (1 mM)	0.4	14.02	6	98.18
17	NFT (1 mM)	0.4	11.68	2.5	95.93

a Reaction conditions: 4-NP/MND/NFT (10 mL) was reduced with NaBH_4_ (0.4 M, 10 mL) using the AgNPs@CS-g-C_3_N_4_-OH nanocomposite as the nanocatalyst in water at ambient temperature.

b No noticeable reaction occurred.

c CS-g-C_3_N_4_-OH matrix support material was used as a catalyst.

d g-C_3_N_4_-OH was used as a catalyst.

e Cross-linked chitosan with glutaraldehyde matrix was used as a catalyst.

f Bare AgNPs were used as a catalyst.

### Substrate Scope Study

The versatility of the AgNPs@CS-g-C_3_N_4_-OH nanocomposite was evaluated using a diverse set of nitroaromatic substrates, including 2-nitrobenzyl alcohol, 4-nitrobenzyl alcohol, 4-nitrobenzaldehyde, 2-nitroaniline, 4-nitroaniline, 2,4-dinitroaniline, 4-nitroanisole, 4-nitrotoluene, 3-nitroacetophenone, and 2,4-dinitrophenol, as displayed in Table 2, **2a**–**2j** compounds, and the corresponding spectra are shown in Figure S4a-j. All substrates were effectively reduced to their corresponding amines under mild aqueous conditions at room temperature, demonstrating the broad substrate tolerance of the catalytic system.^40^ The conversion efficiency strongly depends on the nature and position of substituents.^41^ Substrates such as 2-nitroaniline and 4-nitroaniline exhibited the highest conversion efficacy, which can be attributed to the favorable adsorption and activation of the nitro group on the AgNPs@CS-g-C_3_N_4_-OH nanocomposite surface. Similarly, 4-nitroanisole also showed high conversion, suggesting that the methoxy group does not significantly hinder the reduction process under the present conditions. In contrast, substrates containing carbonyl functionalities, such as 4-nitrobenzaldehyde and 3-nitroacetophenone, displayed comparatively lower conversion efficiencies. This behavior is attributed to the competitive adsorption of carbonyl groups (−CHO and −COCH_3_) on the nanocatalyst surface, thereby reducing the availability of active sites for nitro group reduction.^41,42^ The positional effect is also evident, as 4-nitrobenzyl alcohol shows higher conversion than 2-nitrobenzyl alcohol, indicating that steric hindrance in ortho-substituted substrates limits the accessibility of the nitro group. A similar trend is observed for 4-nitrotoluene, where moderate conversion is achieved due to the methyl group’s electron-donating effect.^41^ Substrates with multiple nitro groups, such as 2,4-dinitroaniline and 2,4-dinitrophenol, exhibited good conversion efficiency due to the strong electron-withdrawing effect, which facilitates reduction.^41^ The above results suggest that both electronic effects and steric factors significantly influence the catalytic performance. The AgNPs@CS-g-C_3_N_4_-OH nanocomposite demonstrated effective catalytic performance across a broad range of nitroaromatic substrates, indicating good substrate compatibility under the investigated reaction conditions.

**Table 2:**
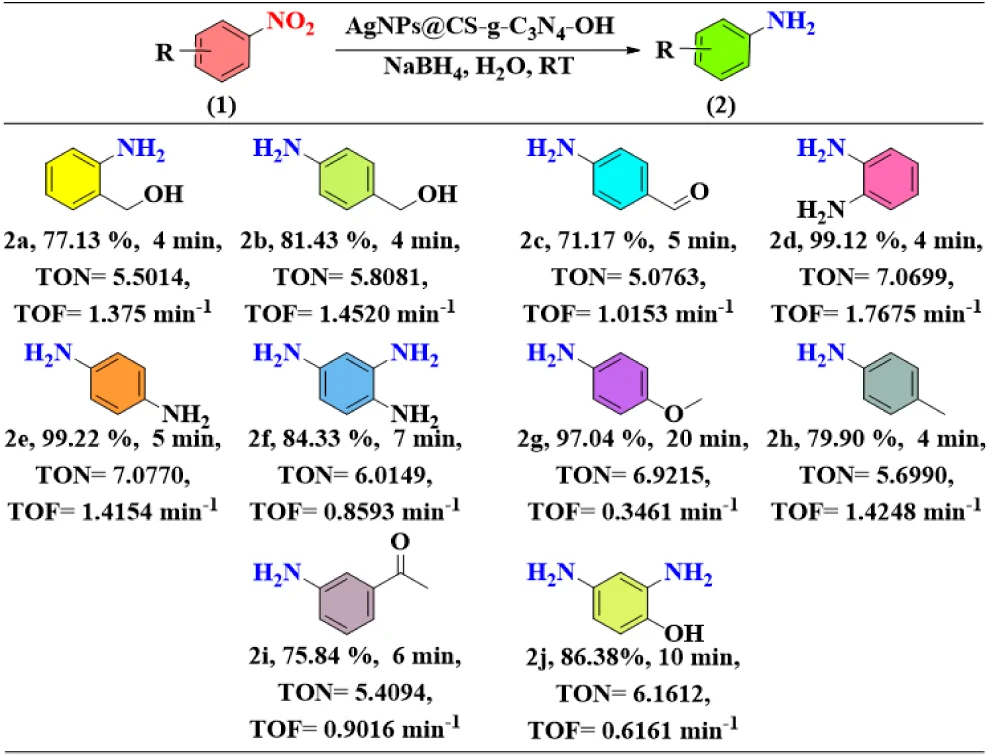
Shows the Reduction of Nitroarenes to Their Corresponding Aromatic Amines Using NaBH_4_ with AgNPs@CS-g-C_3_N_4_-OH Nanocomposite^a^

a Reaction conditions: Nitroarenes (1 mM, 10 mL) were reduced in the presence of aqueous NaBH_4_ (0.4 M, 10 mL) using AgNPs@CS-g-C_3_N_4_-OH nanocomposite (14.02 mol %) as the nanocatalyst at room temperature.

### Plausible Mechanism for the Reduction/Degradation of Nitroaromatics Using AgNPs@CS-g-C_3_N_4_-OH

Scheme S1 illustrates a plausible mechanism for the catalytic reduction of nitroaromatic compounds (4-NP, MND, and NFT) using the AgNPs@CS-g-C_3_N_4_-OH nanocomposite. The high catalytic efficacy of the AgNPs@CS-g-C_3_N_4_-OH nanocomposite toward nitro compounds can be rationalized within the framework of the Langmuir–Hinshelwood mechanism. In the 4-NP system, NaBH_4_ is expected to act as the hydride donor, while the nitro (−NO_2_) group serves as the electron acceptor. In alkaline media, NaBH_4_ releases reactive hydride species; however, direct reduction is kinetically hindered because the system lacks an efficient kinetic pathway for electron or hydride transfer in homogeneous solution, likely owing to a substantial mismatch in potential energy between the donor and acceptor.^2^ The AgNPs present on the CS-g-C_3_N_4_-OH matrix are proposed to overcome this barrier by providing primary catalytic active sites for adsorption and electron transfer, thereby enabling the transfer of hydride ions from BH_4_^–^ to the nitro group. When both (BH_4_^–^ and 4-nitrophenolate) species adsorb onto the AgNPs surface, the nanoparticles may plausibly mediate hydrogen transfer in the form of hydride-like species (H^–^ or coupled electron and proton pairs) from one region of the surface, while simultaneously donating electrons to the bound nitrophenolate at another site. This mechanism is proposed to cooperatively drive the stepwise reduction of the nitro group through nitroso and hydroxylamine intermediates, ultimately yielding the corresponding amine derivative.^20,43^ Within this proposed framework, the Fermi level of AgNPs may effectively serve as a shared electrochemical reservoir, enabling this sequential transformation. Following reduction, the products are expected to desorb from the catalyst surface, regenerating active sites for further turnover cycles. The synergistic interaction between AgNPs and the support contributes to the enhanced catalytic efficiency and recyclability across different nitroaromatic substrates. The CS-g-C_3_N_4_-OH matrix may help to stabilize the AgNPs on the support surface via coordination of Ag through its amine and hydroxyl functional groups. Additionally, the hydroxylated g-C_3_N_4_ acts as an electron-conducting support with a conjugated π-electron network that facilitates rapid electron transfer to the AgNPs (catalytic centers) and suppresses electron recombination, improving the efficiency of electron transfer during the reduction reaction. Since MND and NFT are also nitro-containing substrates that are expected to undergo NaBH_4_-assisted reduction through the same AgNPs-mediated mechanism, the catalytic activity observed in this study is attributed to the presence of AgNPs rather than the support material.

### AgNPs Leaching Study

The leaching of AgNPs from the AgNPs@CS-g-C_3_N_4_-OH nanocomposite was studied to assess its intrinsic heterogeneity, evaluate its stability, and eliminate the potential risk of cross-contamination and ecosystem disruption. Accordingly, the leaching of AgNPs was evaluated and quantified by a hot filtration test and ICP-MS analysis of the filtrate obtained from the reaction mass under the optimized reaction conditions for the reduction of 4-NP to 4-AP. The reaction reached a conversion of 85.58% after 4 min, which is half of the optimized reaction time of 8 min. At this point, the AgNPs@CS-g-C_3_N_4_-OH nanocomposite was removed by filtration. The filtrate was allowed to react for another 4 min under identical conditions. The final conversion after 8 min was 94.18%, representing only a modest increase of 8.60% following catalyst removal. The small increase in conversion efficiency indicates that most of the catalytic activity comes from the AgNPs, while the contribution from dissolved AgNPs is minimal. Following this, the recovered AgNPs@CS-g-C_3_N_4_-OH nanocomposite was subjected to ICP-MS analysis, which revealed a silver concentration of ∼0.3 ppm, corresponding to negligible metal leaching of 3.75%. This minimal leaching indicates that the AgNPs are strongly immobilized on the CS-g-C_3_N_4_-OH surface and remain largely intact during the catalytic processes, although a fraction of smaller nanoparticles may have dissolved and leached from the support. This indicates strong metal–support interactions and confirms the good stability of the catalyst under the optimized reaction conditions.

### Comparative Evaluation

A comparative evaluation of the catalytic performance of the AgNPs@CS-g-C_3_N_4_-OH nanocomposite was carried out against various reported Ag-based nanocatalysts for the reduction of nitroaromatic compounds including 4-NP, MND, and NFT. The results are summarized in Table 3, clearly demonstrating the superior catalytic efficiency of the AgNPs@CS-g-C_3_N_4_-OH nanocomposite. These findings highlight the strong potential as an effective and practical catalyst for the removal of toxic nitroaromatic pollutants from aqueous environments.

**Table 3:** Comparative Analysis of Catalytic Efficiency of AgNPs@CS-g-C_3_N_4_-OH Nanocomposite with Representative Ag-Based Nanocatalysts Reported in the Literature for the Reduction of Nitroaromatic Compounds (4-NP, MND, and NFT) under Similar Reaction Conditions

Substrate	Catalyst	Catalyst loading	Time (min)	TON	TOF (min^–1^)	*K*_nor_ (s^–1^g^–1^)	*k*_app_ of total catalyst (s^–1^ g^–1^)	*k*_app_ of Ag (x10^3^ s^–1^ g_Ag_^–1^)	refs
4-NP	Ag/PSZN-5	20 μL	3.33	-	-	0.86	17.29	-	^44^
	Ag0@CB	15 mg	7	-	-	1.08	16.16	-	^45^
	Ag NP/MTP	40 μL	13.2	-	-	0.07	2.76	-	^46^
	AgNPs/pulp	5 mg	2.5	-	-	4.98	24.9	-	^47^
	AgNPs@CS-g-C_3_N4-OH	900 mg L^–1^	8	7.12	0.8897	0.7831	0.0144 ± 0.003248	5.92	Present work
MND	ZFO@Ag-10	10 mg L^–1^	30	-	-	1.53	15.31	-	^48^
	Ag/Fe_3_O_4_-BNT	0.25 g L^–1^	90	-	-	65.32	16.33	-	^4^
	H05 (Halloysite-Fe_3_O_4_-Ag)	2 g L^–1^	60	-	-	8.26	16.5	-	^49^
	AgNPs@CS-g-C_3_N_4_-OH	900 mg L^–1^	6	7.00	1.1617	0.6488	0.0289 ± 0.008705	11.50	Present work
NFT	AgNPs@CS-g-C_3_N_4_-OH	750 mg L^–1^	2.5	8.21	3.2853	1.5114	0.02164 ± 0.00212	10.30	Present work

### Recyclability

The recyclability study of the AgNPs@CS-g-C_3_N_4_-OH nanocomposite is shown in Figure 6a–c. Figure 6a shows that the AgNPs@CS-g-C_3_N_4_-OH nanocomposite retains high conversion efficacy over multiple cycles (up to 5 cycles) with only a slight decrease in activity (99.89%, 99.81%, 99.81%, 99.29%, 99.23%, and 99.01%), indicating good stability and reusability. This minor loss is likely due to partial surface fouling or limited blockage of active sites by the slight structural modification. The AgNPs@CS-g-C_3_N_4_-OH nanocomposite exhibited a recovery of ∼84.79%, indicating minimal loss during the separation process and confirming its recyclability. PXRD analysis of the fresh catalyst shows characteristic peaks of AgNPs@CS-g-C_3_N_4_-OH nanocomposite along with distinct reflections of corresponding planes of fcc AgNPs, confirming their successful incorporation. After reuse, as revealed in Figure 6b, the recycled catalyst exhibits no noticeable shift in peak positions, and the reduced intensity suggests minor nanoparticle aggregation or a small decrease in crystallinity, without the formation of any new phases. FE-SEM analysis of the fresh catalyst revealed a rough, layered, and porous morphology with well-dispersed AgNPs over the CS-g-C_3_N_4_-OH matrix. In comparison, the recycled catalyst, as depicted in Figure 6c, maintains a similar structural framework but shows slight agglomeration and surface densification, which may reduce the accessibility of active sites. Figure 6d represents the TEM image of the reused AgNPs@CS-g-C_3_N_4_-OH nanocomposite, showing that the nanoparticles are more widely distributed on the surface compared to the fresh catalyst and exhibit an increased particle size, indicating that the catalyst underwent slight structural modification. The particle-size distribution of the used AgNPs@CS-g-C_3_N_4_-OH nanocomposite (Figure 6e) shows an increase in the average AgNP size from 12.82 ± 6.33 nm to 30.16 ± 10.73 nm (*N* = 100) after repeated catalytic cycles. This broader size distribution may be attributed to nanoparticle growth and partial agglomeration. Collectively, the combined results from Figure 6a–e confirm that the catalyst maintains its structural integrity after repeated use, and the slight decline in activity is mainly attributed to surface effects rather than material degradation, demonstrating its robustness for catalytic applications.

**Figure 6:**
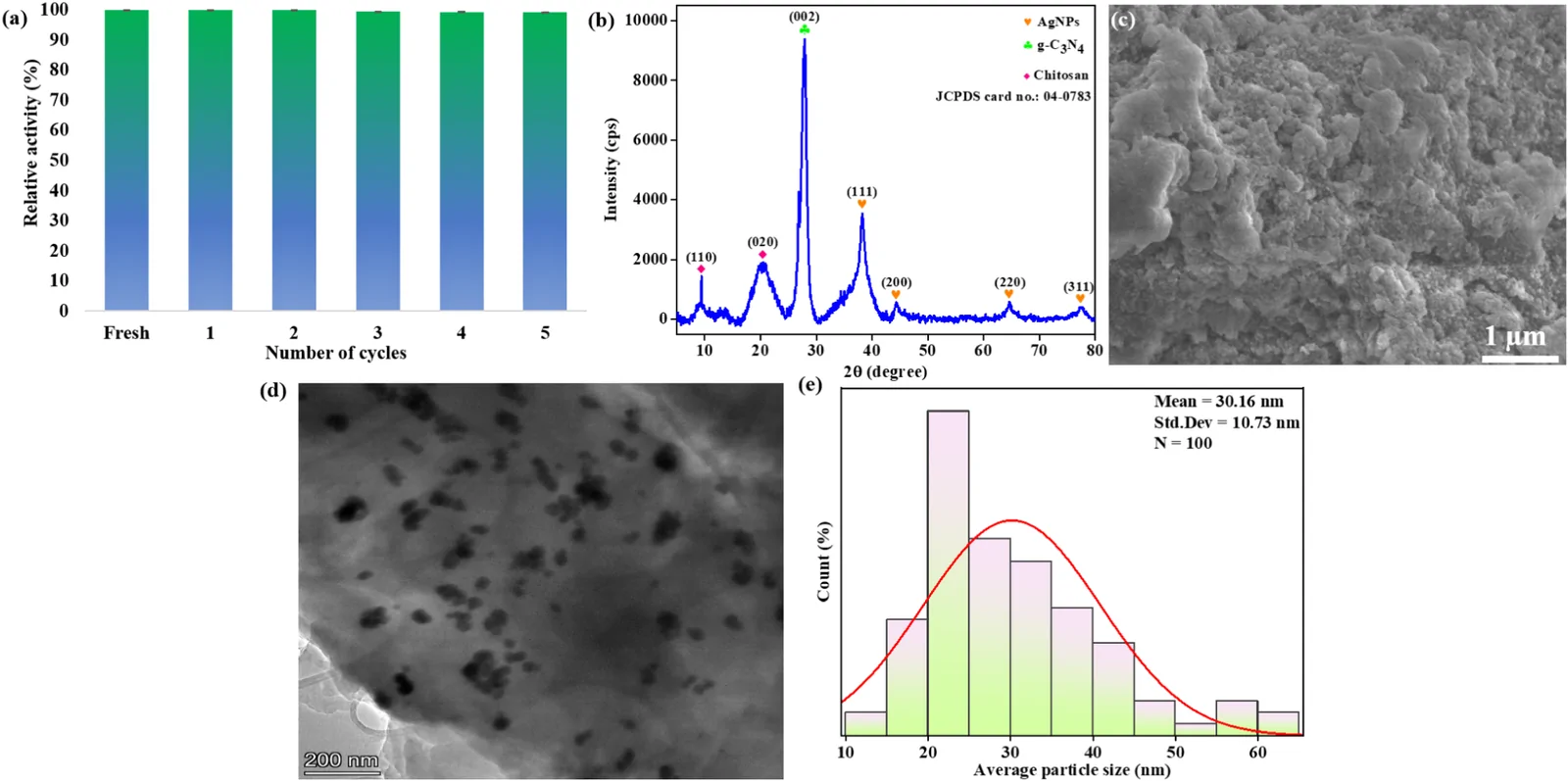
Recyclability assessment of the AgNPs@CS-g-C_3_N_4_-OH nanocomposite for the catalytic reduction of 4-NP: (a) relative activity; (b) PXRD patterns of the recycled AgNPs@CS-g-C_3_N_4_-OH nanocomposite; (c) FE-SEM image of the recycled AgNPs@CS-g-C_3_N_4_-OH nanocomposite; (d) TEM image of reused AgNPs@CS-g-C_3_N_4_-OH nanocomposite; and (e) average particle size distribution of reused AgNPs@CS-g-C_3_N_4_-OH nanocomposite.

### Antibacterial Activity by Agar Well Diffusion Assay

The antibacterial efficacy of the test samples (CS-g-C_3_N_4_-OH and AgNPs@CS-g-C_3_N_4_-OH nanocomposite) was assessed against *A. hydrophila* and *E. faecalis* using the agar well diffusion method. The results (Figure 7a–d and Table 4) demonstrate a concentration-dependent increase in the zones of inhibition (ZOI). As shown in Figure 7a, CS-g-C_3_N_4_-OH exhibited moderate antibacterial activity against *A. hydrophila*, producing a maximum inhibition zone of 10.85 ± 0.21 mm at 500 μg mL^–1^, while no inhibition was observed at lower concentrations (50 and 100 μg mL^–1^). Similarly, against *E. faecalis* (Figure 7b), CS-g-C_3_N_4_-OH showed limited activity, with measurable inhibition only at the highest concentration (13.85 ± 0.21 mm). In contrast, the AgNPs@CS-g-C_3_N_4_-OH nanocomposite displayed enhanced antibacterial activity (Figure 7d), particularly against *E. faecalis*, with inhibition zones of 16.45 ± 0.35 mm at 500 μg mL^–1^ and measurable activity even at lower concentrations (Table 4). Additionally, the AgNPs@CS-g-C_3_N_4_-OH nanocomposite demonstrated consistent inhibition against *A. hydrophila*, as shown in Figure 7c, with zones ranging from 13.45 ± 0.35 to 17.1 ± 0.56 mm across tested concentrations. The positive control (gentamicin) showed the highest inhibition (24–25 mm), validating the experimental conditions. Overall, AgNPs@CS-g-C_3_N_4_-OH nanocomposite exhibited good antibacterial activity compared with the CS-g-C_3_N_4_-OH matrix, suggesting stronger bioactive potential.

**Figure 7:**
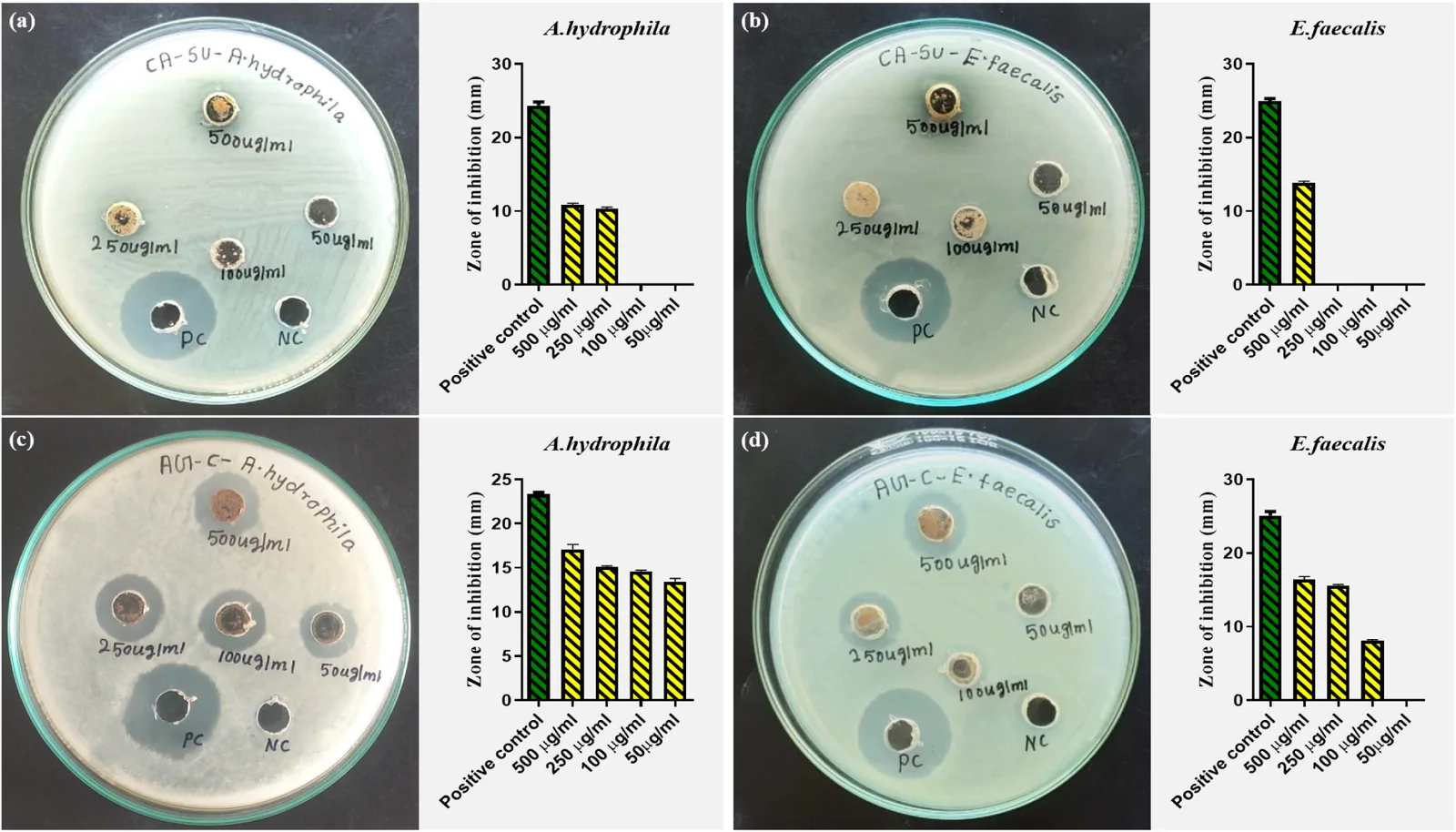
Agar well diffusion assay images and corresponding zone of inhibition (ZOI) bar graphs of (a, b) CS-g-C_3_N_4_-OH and (c, d) AgNPs@CS-g-C_3_N_4_-OH nanocomposite against *A. hydrophila* (MTCC-7966) and *E. faecalis* (MTCC-439) at concentrations of 50, 100, 250, and 500 μg mL^–1^. PC = positive control (gentamicin); NC = negative control.

**Table 4:** Mean Zone of Inhibition (mm) ± Standard Deviation Obtained by CS-g-C_3_N_4_-OH and AgNPs@CS-g-C_3_N_4_-OH Nanocomposite against *A. hydrophila* and *E. faecalis*

			Zone of inhibition (mm) SD ± mean				
S. No.	Name of the test sample	Name of the test organism	500 μg mL^–1^	250 μg mL^–1^	100 μg mL^–1^	50 μg mL^–1^	PC
1.	CS-g-C_3_N_4_-OH	*A. hydrophila*	10.85 ± 0.2	10.35 ± 0.2	0	0	24.35 ± 0.5
		*E. faecalis*	13.85 ± 0.2	0	0	0	24.95 ± 0.4
2.	AgNPs@CS-g-C_3_N_4_-OH	*A. hydrophila*	16.45 ± 0.4	15.60 ± 0.1	8.1 ± 0.1	0	25.1 ± 0.6
		*E. faecalis*	17.10 ± 0.7	15.10 ± 0.1	14.6 ± 0.1	13.45 ± 0.4	23.5 ± 0.2

### Minimum Inhibitory Concentration for 50% Growth Inhibition (MIC_50_) Assay

The MIC values of AgNPs@CS-g-C_3_N_4_-OH nanocomposite against *A. hydrophila* and *E. faecalis* were determined using the broth microdilution method. An observable antibacterial effect was first detected at a concentration of 0.9 μg mL^–1^ for both strains. The dose–response relationship, which is displayed in Figure S5 in the Supporting Information, is evident from the OD and percentage inhibition data, in which increasing concentrations of the AgNPs@CS-g-C_3_N_4_-OH nanocomposite led to a progressive decrease in bacterial growth. As shown in Figure 8a–d, the percentage inhibition reached approximately 75.92% for *A. hydrophila* and 80.35% for *E. faecalis* at 500 μg mL^–1^. The MIC_50_ values further confirmed differential susceptibility between the organisms. The MIC_50_ for *A. hydrophila* was 28.45 μg mL^–1^, whereas *E. faecalis* showed a lower MIC_50_ of 13.40 μg mL^–1^, indicating higher sensitivity. The regression analysis yielded high goodness-of-fit values (*R* ^2^ = 0.9849 for *A. hydrophila* and 0.9765 for *E. faecalis*), supporting the reliability of the results.

**Figure 8:**
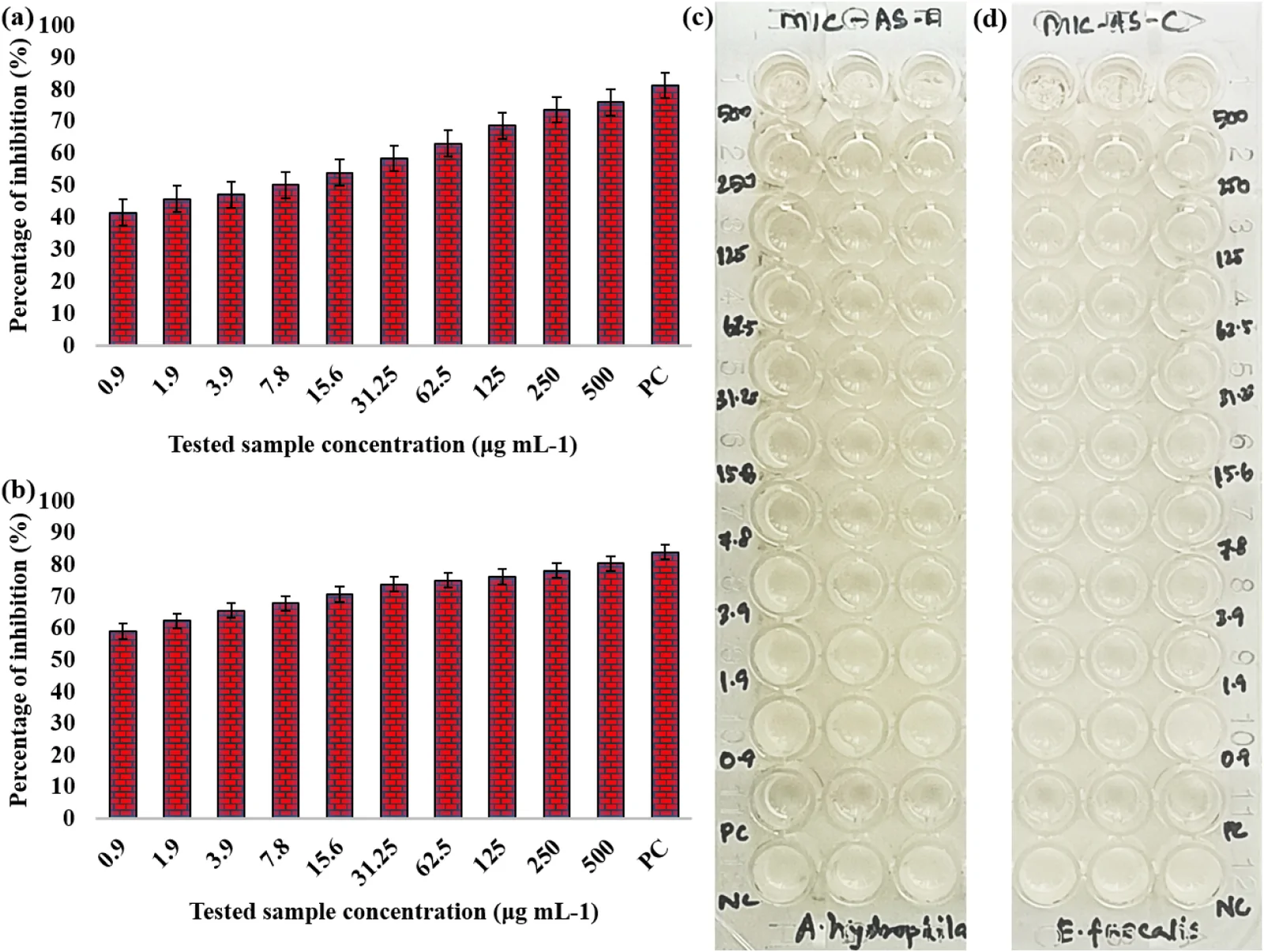
(a, b) Bar graphs showing percentage inhibition and (c, d) photographic images of 96-well microtiter plates illustrating the minimum inhibitory concentration (MIC) of AgNPs@CS-g-C_3_N_4_-OH nanocomposite against *A. hydrophila* and *E. faecalis*. PC = positive control; NC = negative control.

### Antioxidant Activity

The DPPH assay revealed a concentration-dependent radical scavenging activity of the AgNPs@CS-g-C_3_N_4_-OH nanocomposite, as presented in Figure 9a and b. The control experiment exhibited a mean absorbance (OD) of 1.212, whereas the absorbance decreased significantly with increasing concentration of the sample, indicating effective quenching of DPPH radicals. At the highest tested concentration (500 μg mL^–1^), the AgNPs@CS-g-C_3_N_4_-OH nanocomposite demonstrated a scavenging activity of 71.09%, which gradually decreased to 39.52% at 10 μg mL^–1^. This trend confirms that the antioxidant activity is directly proportional to the concentration of the sample. In comparison, ascorbic acid showed a significantly higher inhibition of 96.24%, demonstrating its superior radical scavenging efficiency. The IC_50_ value of the AgNPs@CS-g-C_3_N_4_-OH nanocomposite was calculated to be 104.6 μg mL^–1^, indicating moderate antioxidant activity. The dose–response curve showed good fitting with a regression coefficient (*R* ^2^) of 0.9675, confirming the reliability of the experimental data and analysis. The observed sigmoidal behavior with a negative Hill slope (−1.674) further supports a typical radical scavenging mechanism involving cooperative interactions. The antioxidant activity of the AgNPs@CS-g-C_3_N_4_-OH nanocomposite can likely be attributed to its ability to participate in both hydrogen atom transfer (HAT) and single electron transfer (SET) processes.^50^ The presence of functional groups such as hydroxyl and amine moieties within the matrix facilitates hydrogen donation, while the nanostructured framework may promote electron transfer reactions. Additionally, the material’s surface provides active sites for the adsorption and stabilization of the DPPH radical, thereby enhancing the overall scavenging efficiency. The material’s ability to scavenge free radicals also suggests its role in mitigating oxidative stress, a crucial process in biological systems. The antioxidant activity observed for the AgNPs@CS-g-C_3_N_4_-OH nanocomposite reflects the synergistic effect of the integrated nanocomposite. g-C_3_N_4_-OH, chitosan, and AgNPs have each been reported to possess radical-scavenging capacity attributed to distinct functional groups and surface chemistry. However, the individual contribution of each constituent was not experimentally investigated in the present study, representing a limitation. Further studies involving comparative antioxidant evaluation of the individual components and detailed mechanistic investigations will be necessary to establish their specific contributions.

**Figure 9:**
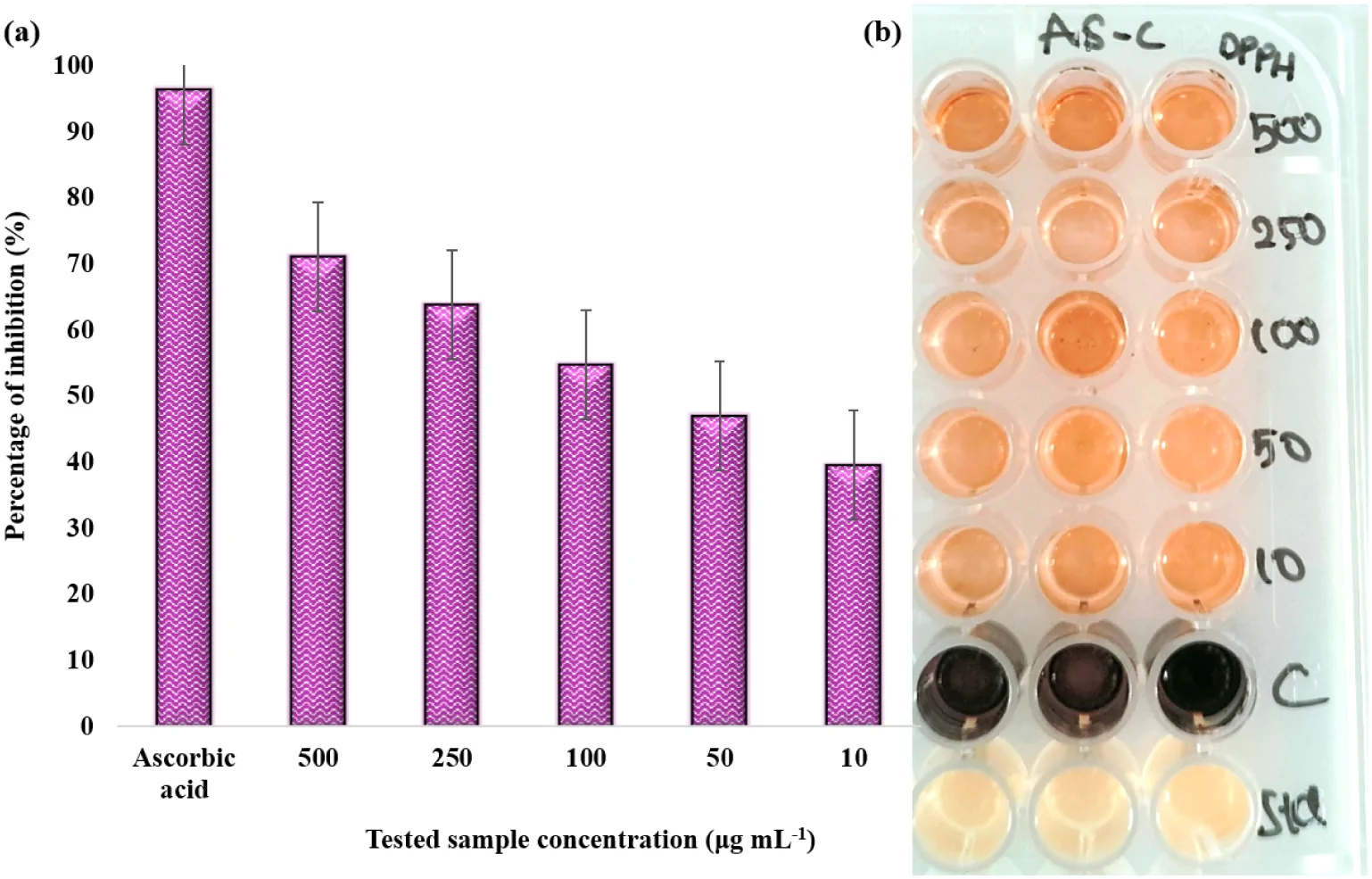
(a) Bar graph showing concentration-dependent DPPH radical scavenging activity (%) and (b) photographic image of the corresponding 96-well plate illustrating the color change from dark purple to pale orange of AgNPs@CS-g-C_3_N_4_-OH nanocomposite. C = control; Std = ascorbic acid (standard).

## Conclusions

In conclusion, the AgNPs@CS-g-C_3_N_4_-OH nanocomposite was effectively constructed through a mild and convenient approach, in which the functional groups of the chitosan-g-C_3_N_4_-OH framework enabled both the reduction and stabilization of AgNPs. The material exhibited a well-integrated structure with crystalline silver and a stable support matrix. Functionally, the composite demonstrated rapid and efficient catalytic reduction of nitro compounds, achieving 99.79%, 97.91%, and 95.93% conversion for 4-NP, MND, and NFT, respectively, with highrate constants (0.9871–0.9774 min^–1^) and short half-lives (∼0.70 min). Its consistent performance across multiple cycles highlights its durability and practical relevance. Biologically, the material showed strong antibacterial efficacy. The MIC_50_ values (28.45 and 13.40 μg mL^–1^) further confirm its potency. In addition, moderate antioxidant activity (IC_50_ = 104.6 μg mL^–1^; 71.09% inhibition) suggests auxiliary biomedical potential. Overall, this multifunctional nanocomposite combines efficient catalysis, moderate antibacterial action, and antioxidant capability under the tested conditions, making it a promising candidate for environmental and biomedical applications.

## Supplementary Material



## Notes

Figures S1 to S5, Scheme S1, and equations S1 and S2 (PDF)
