# Supplementary material for: Multifunctional AgNPs@Chitosan-g‑C3N4‑OH Nanocomposite: Efficient Catalytic Reduction of Nitro Pollutants, Antibacterial and Antioxidant Applications
Source: ACS Omega. 2026 Oct 5;11(41):62700–16. doi: 10.1021/acsomega.6c04143 (PMC13688489; doi:10.1021/acsomega.6c04143)
Supplement: Supplementary file 1 [file ao6c04143_si_001.pdf]

Supporting Information

Multifunctional AgNPs@Chitosan-g-C_3N_4-OH nanocomposite: Efficient catalytic reduction of nitro pollutants, antibacterial, and antioxidant applications

Aakhila Banu A[a], Abhishek Kumar[b], Channabasaveshwar V Yelamaggad[b], Bhari Mallanna Nagaraja[a], Alejandro Bugarin[c]*, Siddappa A. Patil[a,c]*

[a]*Centre for Nano and Material Sciences, Jain (Deemed-to-be University), Jain Global Campus, Kanakapura, Ramanagaram, Bangalore 562112, India.*

[b]*Centre for Nano and Soft Matter Sciences, Survey No. 7, Shivanapura, Bangalore, 562162, India.*

[c]*Department of Chemistry & Physics, Florida Gulf Coast University, Fort Myers, FL 33965, USA.*

This Supporting Information contains the following details:

Number of Figures: 5

Number of Schemes: 1

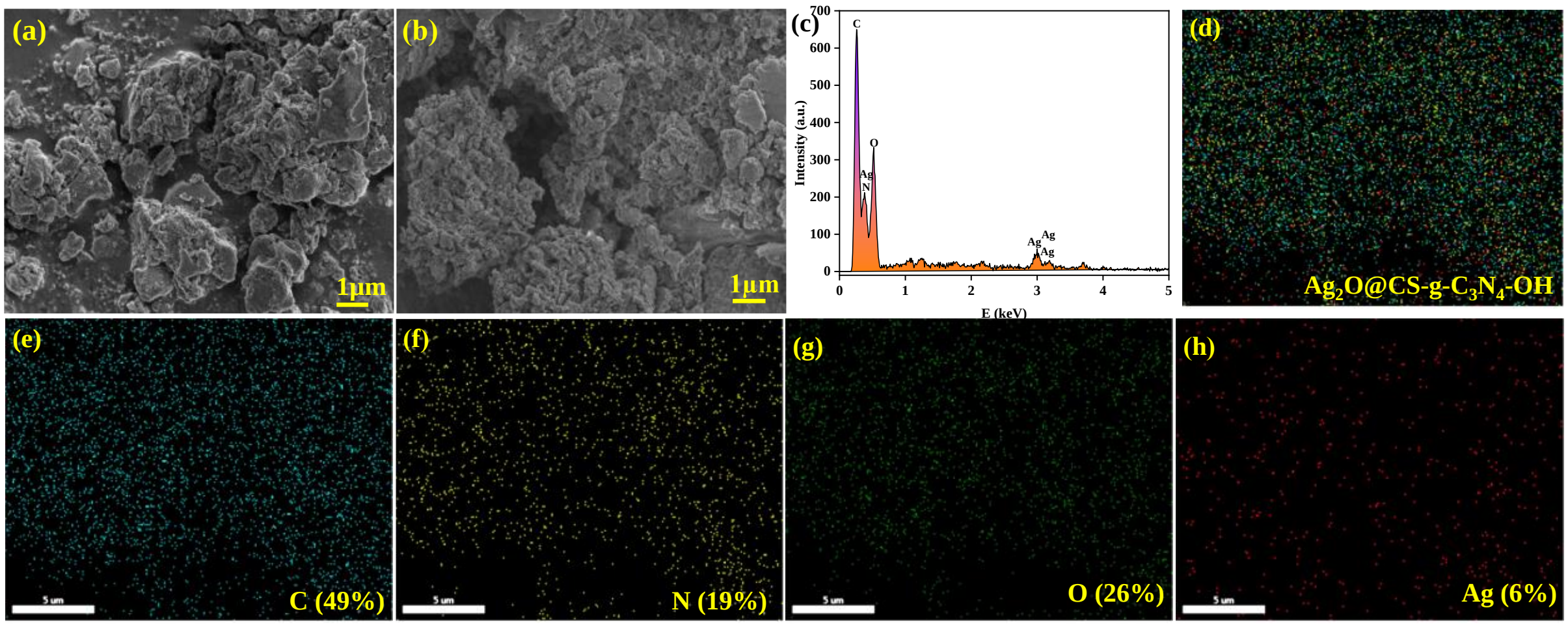

Figure S1. The FE-SEM images of (a) CS-g-C_3N_4-OH and (b) AgNPs@CS-g-C_3N_4-OH nanocomposite; EDS spectrum of (c) AgNPs@CS-g-C_3N_4-OH nanocomposite; and elemental mapping of (d-h) AgNPs@CS-g-C_3N_4-OH nanocomposite.

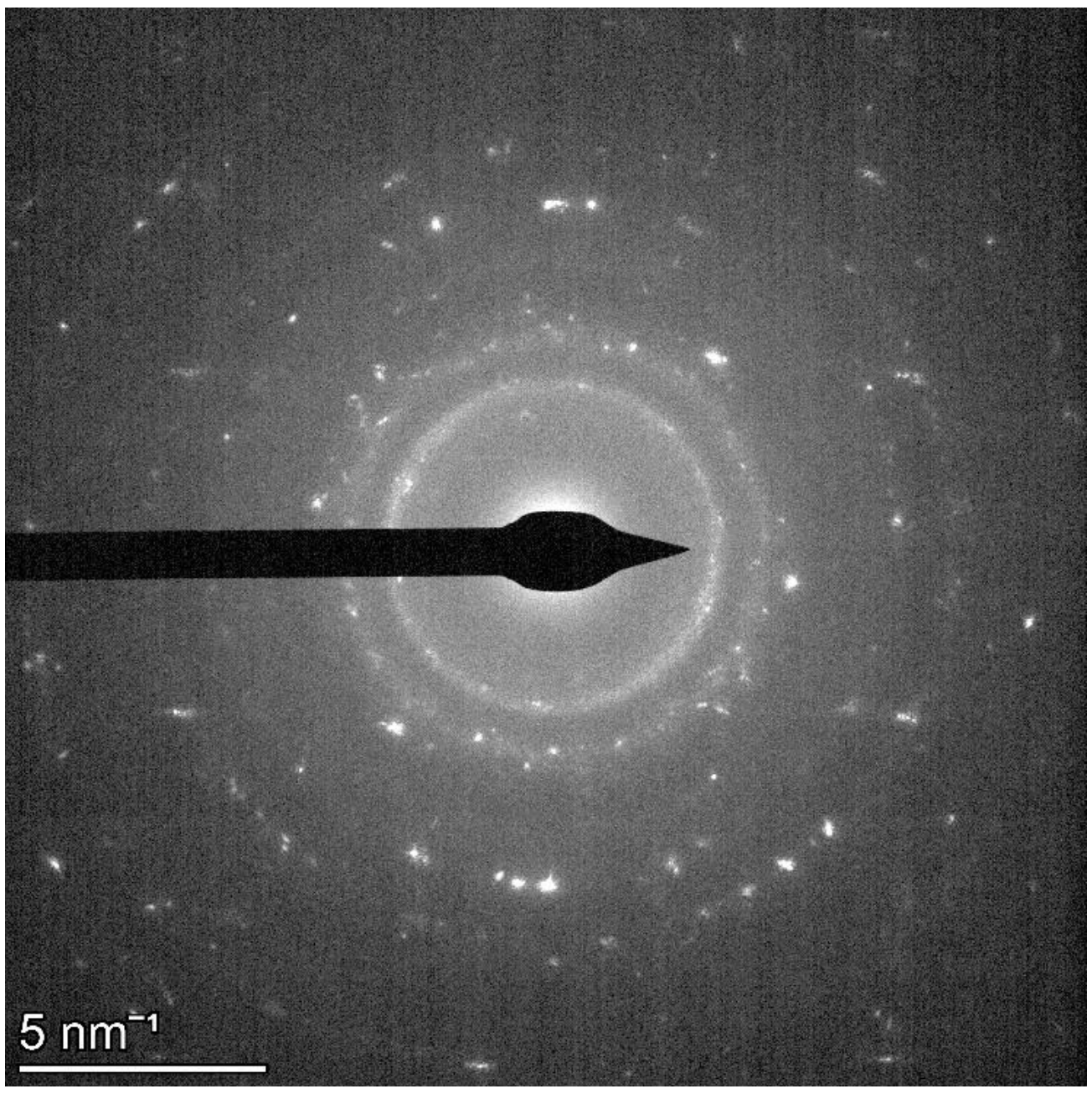

Figure S2. Raw SAED image of the AgNPs@CS-g-C_3N_4-OH nanocomposite.

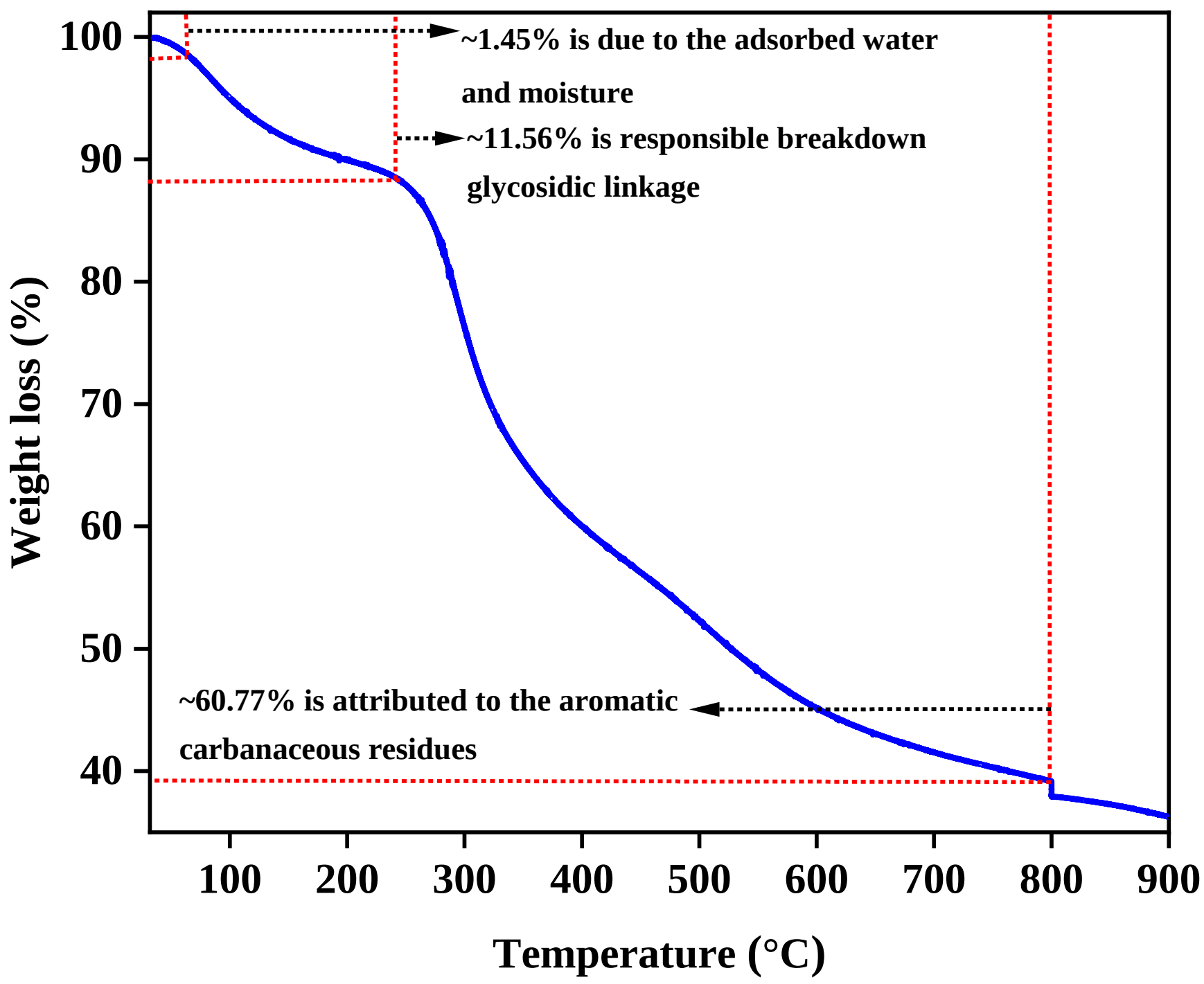

Figure S3. TGA curve of the AgNPs@CS-g-C_3N_4-OH nanocomposite.

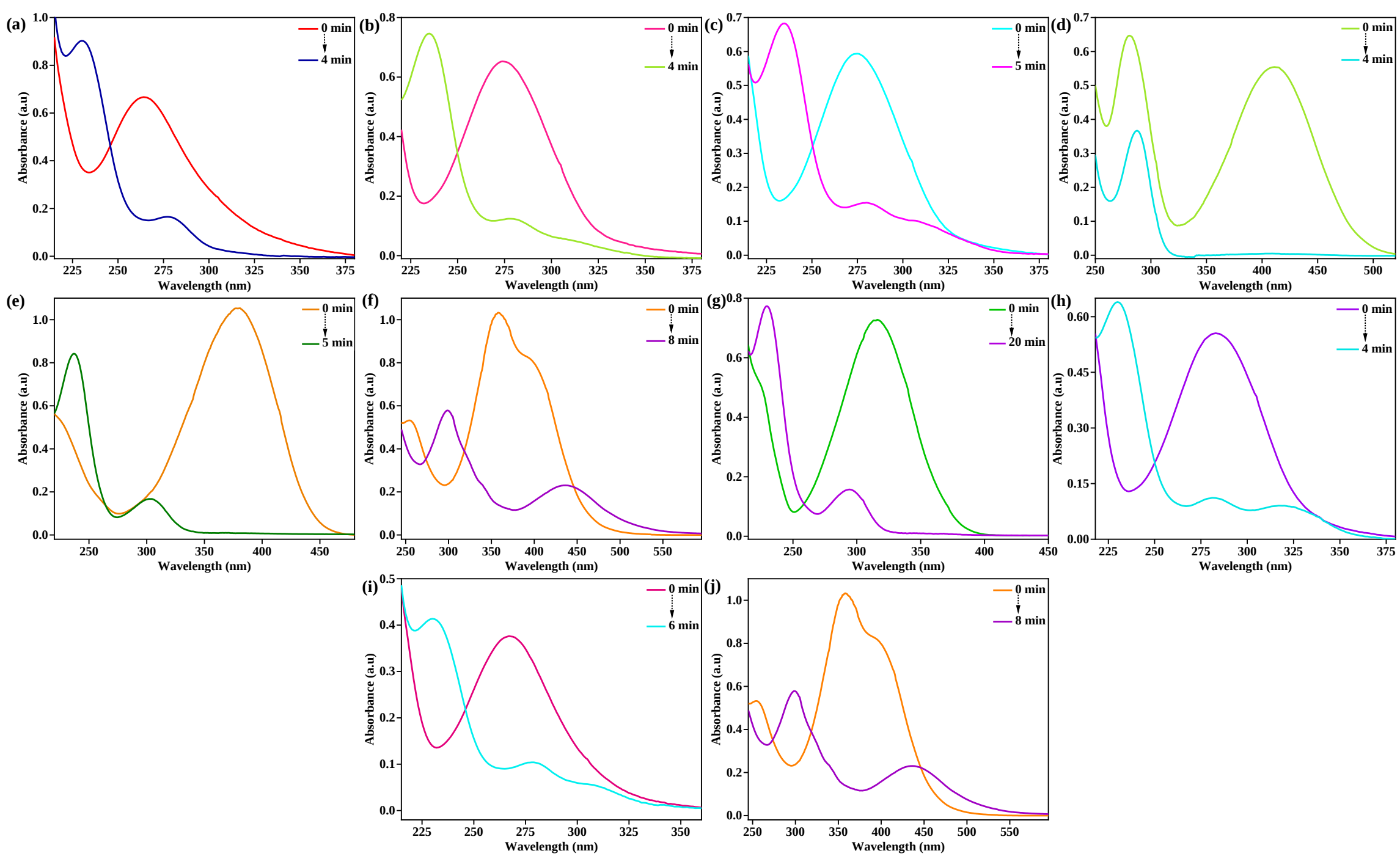

Figure S4. Time-dependent UV-Visible absorption spectra showing the catalytic reduction of nitroaromatic compounds, such as (2a) 2-nitrobenzyl alcohol, (2b) 4-nitrobenzyl alcohol, (2c) 4-

nitrobenzaldehyde, (2d) 2-nitroaniline, (2e) 4-nitroaniline, (2f) 2,4-dinitroaniline, (2g) 4-nitroanisole, (2h) 4-nitrotoluene, (2i) 3-nitroacetophenone, and (2j) 2,4-dinitrophenol in the presence of AgNPs@CS-g-C_3N_4-OH nanocomposite.

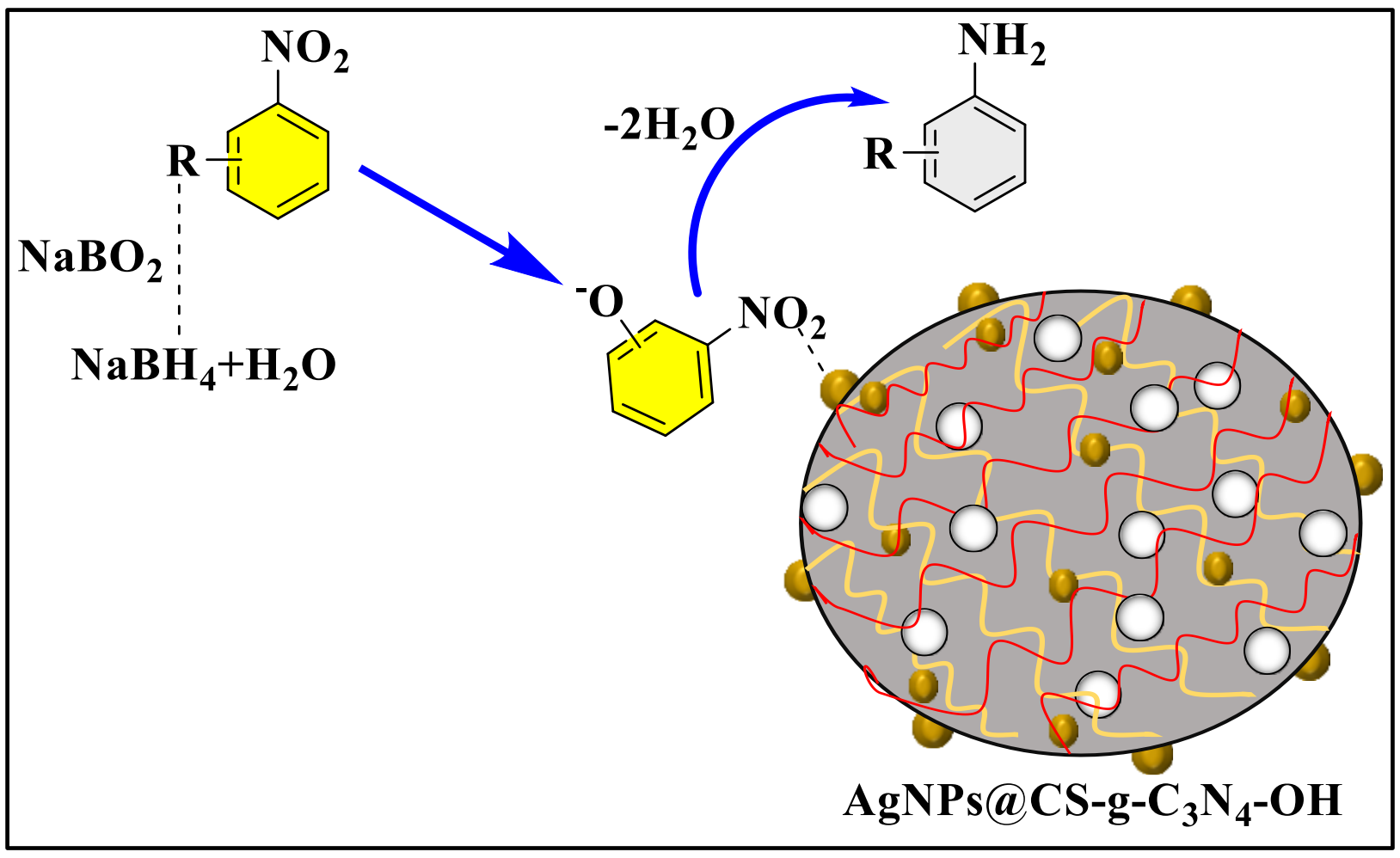

Scheme S1. Plausible reaction pathway for the catalytic reduction of nitro aromatics to their corresponding amines mediated by the AgNPs@CS-g-C_3N_4-OH nanocomposite.[1]

First-order rate equation:

$$\ln\left(\frac{C_t}{C_0}\right) = -kt \text{...(S1)}$$

Where C_0 is the initial concentration and absorbance, C_t is the concentration and absorbance at time t, and k is the rate constant.

Equation for % conversion

$$\%\ \text{conversion} = \left(\frac{C_0 - C_t}{C_0}\right) \text{x } 100 \text{...(S2)}$$

C_0 and C_t are the initial absorbance and the absorbance at time t.

Example (Table 1, entry 8):

C_0 = 1.3442 and C_t = 0.0027 at t = 8 min

$$\%\ \text{conversion} = \left(\frac{1.3442 - 0.0027}{1.3442}\right) \text{x } 100 = 99.79\ \%$$

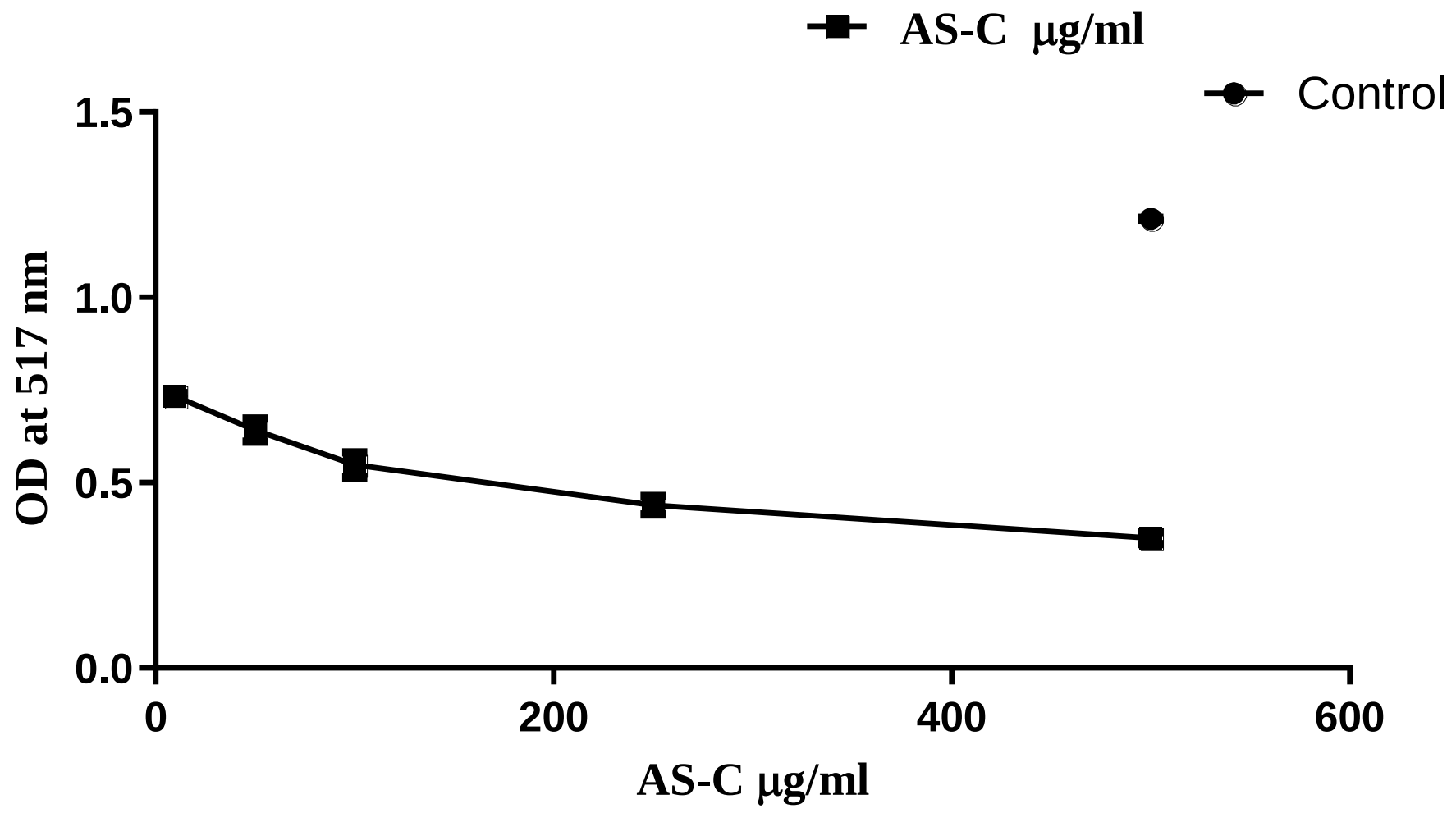

Figure S5. Dose-response curve of AgNPs@CS-g-C_3N_4-OH nanocomposite for DPPH radical-scavenging activity.

References:

1. a) Banu, A.; Antony, A. M.; Sasidhar, B.; Faisal, M.; Harraz, F. A.; Patil, S. A. *N*-heterocyclic carbene functionalized boehmite-supported silver nanoparticles as nanocatalyst for reduction of environmental pollutants. *Colloid. Surf. A Physicochem. Eng. Asp.* **2024**, *697*, 134372. b) Banu, A.; Yelamaggad, C.; Patil, S. A. Sparse silver loading on chitosan–boron carbon nitride framework: a sustainable route to multifunctional nanomaterials. *RSC Adv.* **2025**, *15* (50), 42740-42760.
